# Synthesis of 1,2,4-Triazole-3-Thiol Derivatives from Thiosemicarbazides and Carboxylic Acids Using Polyphosphate Ester

**DOI:** 10.3390/molecules30224422

**Published:** 2025-11-16

**Authors:** Bogdan A. Tretyakov, Viktoria I. Tikhonova, Svyatoslav Y. Gadomsky, Nataliya A. Sanina

**Affiliations:** 1 Federal Research Center of Problems of Chemical Physics and Medicinal Chemistry RAS, Chernogolovka 142432, Russia; tretyakov@icp.ac.ru (B.A.T.); tikhonova.vi@phystech.edu (V.I.T.); sanina@icp.ac.ru (N.A.S.); 2 Moscow Center for Advanced Studies, 20 Kulakova Str., Moscow 123592, Russia

**Keywords:** 1,2,4-triazole-3-thiols, triazoles, thiosemicarbazide, acylation, cyclodehydration, polyphosphate ester, hydrothermal reactor

## Abstract

Conditions have been established for the direct reaction of thiosemicarbazides with carboxylic acids in the presence of polyphosphate ester (PPE) to synthesize 1,2,4-triazole-3-thiol derivatives. The synthesis involves two consecutive steps: (i) acylation of the thiosemicarbazide with a carboxylic acid in chloroform in the presence of PPE at 90 °C using a hydrothermal reaction vessel, followed by (ii) cyclodehydration of the acylation product by treatment with an aqueous alkali solution. Using this new synthetic approach, 15 derivatives of 1,2,4-triazole-3-thiol were obtained, five of which were synthesized for the first time. The structures of the synthesized compounds were confirmed by NMR spectroscopy and mass spectrometry.

## 1. Introduction

**Figure 1:**
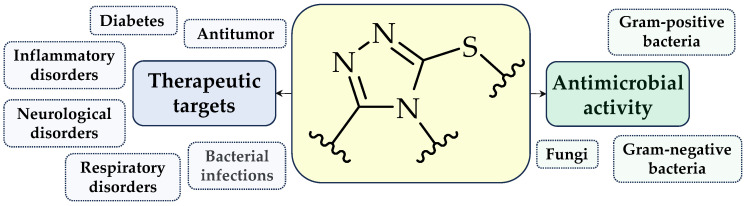
Overview of the biological activities of 1,2,4-triazole-3-thiol derivatives (see Table 1 and Table 2 for specific molecular targets and activity against microbial strains).

Triazoles, in general, hold a special place in modern coordination and medicinal chemistry research [1,2,3,4]. This is also true for such a specific type of triazoles as 1,2,4-triazole-3-thiol derivatives. The biological activity of these compounds has been demonstrated against a range of important targets (Figure 1), though this list is not exhaustive and focuses on the most studied areas. Table 1 shows that 1,2,4-triazole-3-thiols are promising agents for anticancer therapy [5,6,7,8,9,10,11,12,13,14,15,16,17,18,19,20,21,22,23,24,25,26,27], as well as for treating diabetes [28,29,30,31,32,33], inflammatory [34,35,36,37,38], neurological [30,39,40,41,42,43,44,45,46,47,48], and respiratory [35,49,50,51] disorders, and bacterial infections [52,53,54,55,56,57,58]. In addition to their action on molecular targets, there are also examples of antibacterial and antifungal activity [13,59,60,61,62,63,64,65,66] of the discussed triazoles against a range of common strains (Table 2), including pathogens of such dangerous diseases as tuberculosis [63,64,65], pneumonia [60,66], and candidiasis [13,59,60,61].

**Figure 2:**
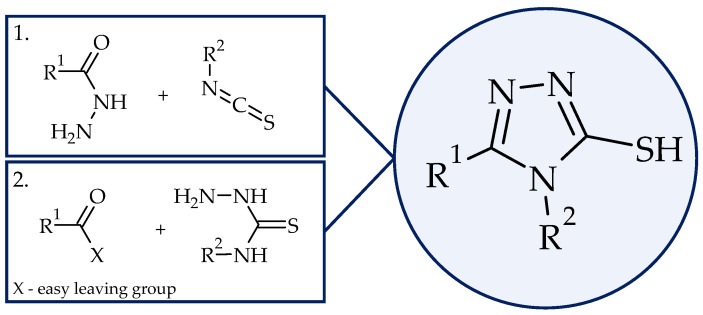
Literature-known approaches to the synthesis of 1,2,4-triazole-3-thiols.

All literature-described approaches to the synthesis of 1,2,4-triazole-3-thiols can be divided into two main types, which are presented as a simplified scheme in Figure 2. The first approach (Figure 2), which involves the reaction of hydrazides with isothiocyanates, serves as the basis for synthesis in most of the works mentioned above [8,9,10,12,13,14,17,18,19,21,22,23,27,30,35,36,37,38,39,41,42,43,46,50,56,58,59,60,61,62,64,65,66]. Thus, in the context of the reviewed literature, this synthetic route is the most prevalent. It is important to note that the first reaction in Figure 2 is presented in a simplified form, as it is not entirely accurate for R^2^ = H. In this case, the reaction is carried out not with thiocyanic acid, but with its salts, i.e., with thiocyanates [9,12,56].

The second approach (Figure 2) involves the acylation of thiosemicarbazide derivatives (with a pre-activated carboxyl group) followed by cyclodehydration under alkaline conditions.

Although this approach is encountered much less frequently in the aforementioned sources [16,20,24,46,47,52,60,63], it offers significantly greater diversity in terms of experimental conditions, which primarily depend on the method of carboxyl group activation. In the vast majority of cases, such reactions have been performed using acid chlorides [24,46,52,60,63]. However, there are also examples employing activating agents such as propylphosphonic anhydride (T3P) [16], HATU (*O*-(7-azabenzotriazol-1-yl)-*N,N,N′,N*′-tetramethyluronium hexafluorophosphate) [20] and 1-ethyl-3-(3′-dimethylaminopropyl)carbodiimide (EDC) [47]. The fact that acid chlorides are most commonly used in the second approach (Figure 2) suggests a high relevance for research aimed at finding more accessible conditions for the acylation of thiosemicarbazides.

**Figure 3:**
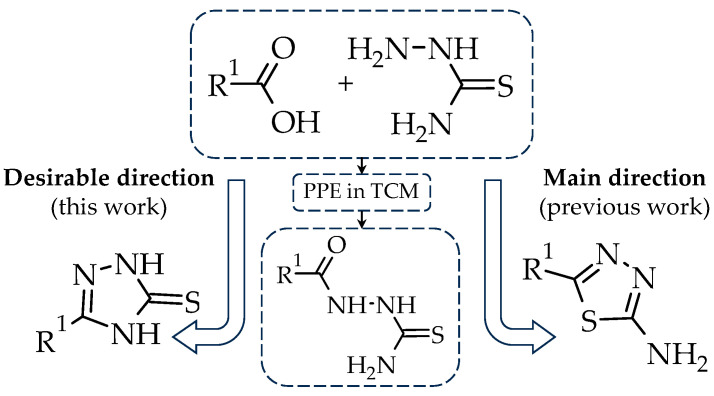
Possible reaction pathways of thiosemicarbazide with carboxylic acids in the presence of PPE.

We have previously shown [67] that in the presence of polyphosphate ester (PPE) the reaction of carboxylic acids with thiosemicarbazide leads to the formation of 1,3,4-thiadiazol-2-amine derivatives. Furthermore, it has been experimentally demonstrated [67], that this synthesis proceeds in two stages with the formation of an intermediate acylation product (Figure 3, main direction), which is a compound that could also be utilized for the synthesis of 1,2,4-triazole-3-thiols. However, how to realize the potential of PPE for the synthesis of the target triazoles remained unclear until now. Therefore, the present study was aimed at finding optimal conditions to shift the course of the reaction between carboxylic acids and thiosemicarbazide in the presence of PPE towards the formation of 1,2,4-triazole-3-thiols (Figure 3).

## 2. Results and Discussion

**Scheme 1:**
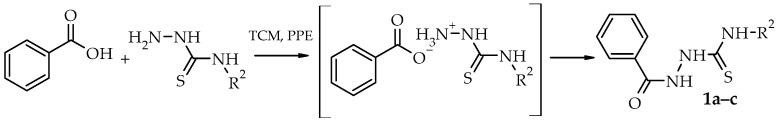
Acylation of thiosemicarbazide derivatives with benzoic acid in the presence of PPE (R^2^ = H, Ethyl, Phenyl).

It was previously shown [67] that the reaction conditions for thiosemicarbazide with benzoic acid in the presence of PPE can be optimized to yield 2-benzoylhydrazine-1-carbothioamide as the main product. However, the universality of these conditions and their applicability to substituted thiosemicarbazides remained unclear. Therefore, reactions of benzoic acid with thiosemicarbazides bearing different substituents (aliphatic and aromatic) were carried out under conditions as close as possible to those described earlier [67] (Scheme 1). Despite an observed trend of decreasing yield (when moving from unsubstituted to aromatic thiosemicarbazide, see descriptions **1a–c** in the Supplementary Materials), the acylation products were isolated and characterized in all three cases. Thus, the principal applicability of PPE for the acylation of various thiosemicarbazides was demonstrated.

The reaction mechanism is no less important. We previously suggested [67] that the reaction proceeds via the intermediate formation of a thiosemicarbazide salt with the carboxylic acid (Scheme 1). To test this hypothesis, we made several attempts to synthesize and isolate the thiosemicarbazide salt with benzoic acid in pure form, intending to subsequently treat it with a solution of PPE in chloroform. However, all attempts to obtain the target salt in aqueous-alcoholic mixtures, as well as in mixtures of water with DMSO and DMF, yielded negative results—only mixtures of the starting compounds could be isolated instead of the target salt. It is quite possible that if the target salt is formed, it hydrolyzes immediately in the presence of even trace amounts of water. Indirect confirmation of this is the following observed feature, which was later implemented in the triazole synthesis method described below: the use of pre-dried chloroform as a solvent significantly increases the yield of the target reaction. Another indirect confirmation of the proposed mechanism (Scheme 1) is the invalidity of a potential alternative viewpoint, suggesting the involvement of a mixed anhydride formed from the carboxylic acid and PPE. The desired acylation reaction does not proceed if the thiosemicarbazide is added to a pre-prepared mixture of the carboxylic acid and PPE (i.e., if the presumed mixed anhydride is allowed to form). The only possible way to achieve the discussed reaction is to pre-mix the starting compounds in dry form followed by treatment with a solution of PPE.

As a result of the search for universal acylation conditions, the following important features were discovered, which later formed the basis of the described method for the synthesis of 1,2,4-triazole-3-thiols: 1. Chloroform is the most universal solvent compared to dichloromethane, chlorobenzene, toluene, ethyl acetate, DMF, and DMSO (in all other solvents, the reaction between benzoic acid and thiosemicarbazide either did not proceed or gave a negligibly low yield of the target compound); 2. During the acylation reaction, the following sequential change in the appearance of the reaction mixture is typically observed: the mixture first becomes homogeneous, followed by the gradual formation of a precipitate of the acylation product (however, in some cases, the product precipitate could form over a week or more); 3. The target 1,2,4-triazole-3-thiols are much easier to isolate in pure form compared to the intermediate acylation products, as they can be readily converted into a water-soluble form as potassium salts (thus, the target product can be separated from impurities by treating the aqueous solution with activated charcoal).

**Scheme 2:**
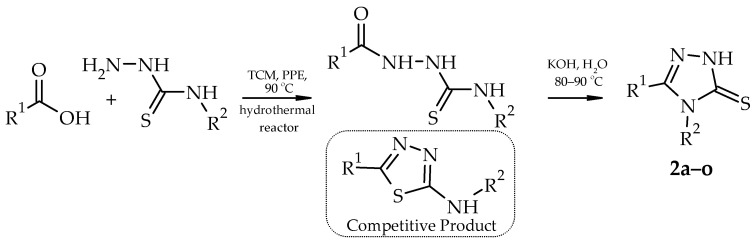
Synthetic scheme used in this work for the preparation of 1,2,4-triazole-3-thiol derivatives (R^1^ and R^2^ values are listed in Table 3).

Considering the features listed above, it was decided to carry out the reaction of carboxylic acids with thiosemicarbazides in two immediate steps (Scheme 2), using a hydrothermal reaction vessel for the first stage. As shown in Scheme 2, the acylation of thiosemicarbazide may form a thiadiazole side product, aligning with the main reaction pathway in the presence of PPE (Figure 3). The two-step method capitalizes on the insolubility of the resulting 1,3,4-thiadiazol-2-amines in alkaline medium to easily separate them from the target 1,2,4-triazole-3-thiols in the second stage. This bypasses the need for intermediate isolation, greatly streamlining the synthesis. The use of a hydrothermal reactor allowed for an increase in the reaction temperature without changing the solvent and accelerated the precipitation of the acylation product from the reaction mixture.

**Figure 4:**
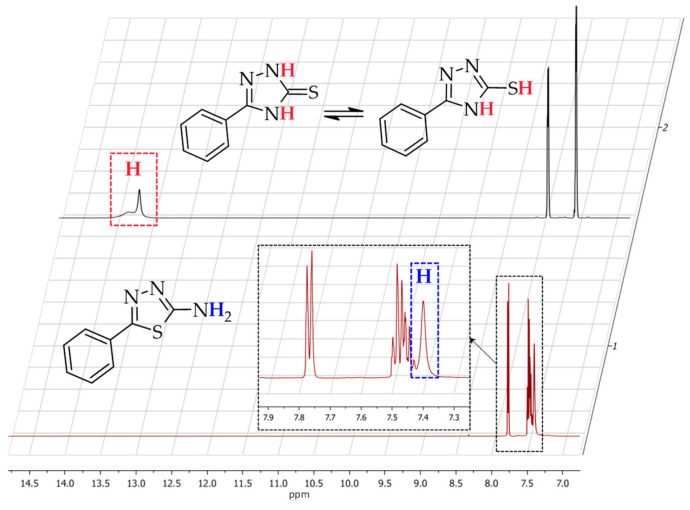
^1^H NMR Spectral Comparison: 5-Phenyl-4*H*-1,2,4-triazole-3-thiol (**top**) and 5-Phenyl-1,3,4-thiadiazol-2-amine (**bottom**).

Given the aforementioned problem of thiadiazole formation as a competitive product, it is also important to discuss the spectral distinctions between 1,2,4-triazole-3-thiol derivatives and their functional isomers, 1,3,4-thiadiazol-2-amines. Figure 4 shows a comparison of the ^1^H NMR spectra of 5-phenyl-4H-1,2,4-triazole-3-thiol and 5-phenyl-1,3,4-thiadiazol-2-amine. A significant difference is evident (Figure 4) in the chemical shift regions for the protons of the triazole and thiadiazole rings. Specifically, the N-H and S-H protons of the triazole resonate at a much lower field (13–14 ppm) compared to the amino group signal of the thiadiazole, which appears in the aromatic region (Figure 4). Thus, it is clear that ^1^H NMR spectroscopy alone is sufficient for the reliable structural identification of the isolated products.

Table 3 presents the results obtained from the implementation of Scheme 2. It can be seen (Table 3) that the yield values are not high; however, it is necessary to consider that these data are calculated for individually isolated compounds after a two-step synthesis. But the most important point to emphasize is the discovered difference between syntheses **2a–h** and **2i–m**. In the first case (**2a–h**), the reaction in the hydrothermal reactor resulted in the formation of an easily separable precipitate of the intermediate, whereas in the case of carboxylic acids with nitrogen-containing heterocycles (**2i–m**), a resin-like mass was formed instead of a precipitate. It is quite probable that in the case of **2i–m**, we observed the formation of a polymeric salt between the quaternized nitrogen of the intermediate (acylated thiosemicarbazide) and the generated residues of polyphosphoric acid.

**Figure 5:**
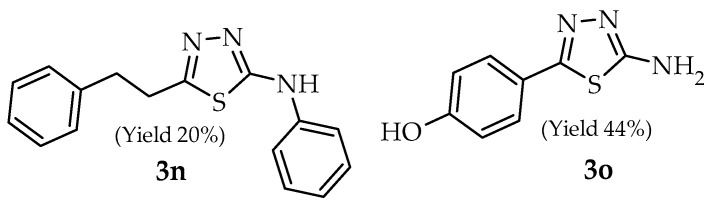
Structures of the reaction products isolated according to Scheme 2 instead of **2n** and **2o**, respectively.

Compounds **2n** and **2o** deserve special attention; their yields are not listed in Table 3. In these cases, using the exact same “hydrothermal” stage conditions as for **2a–m** led to a significant increase in the conversion depth; i.e., the reactions did not stop at the acylation stage but proceeded to the next stage of cyclo-dehydration, forming products **3n** and **3o** (Figure 5). It is noteworthy that in the attempt to synthesize **2n**, after the first stage (Scheme 2), only a trace amount of precipitate was observed (unlike the very similar reactions **2a–h**), and product **3n** was isolated from the remaining chloroform solution of the reaction mixture. Regarding the attempt to synthesize **2o**, similarly to **2i–m**, a resin-like mass was observed after the “hydrothermal” stage, from which product 3o was isolated. This resin formation is most likely associated with the reversible binding of the product’s hydroxyl group with residues of PPE or the forming polyphosphoric acid.

**Scheme 3:**
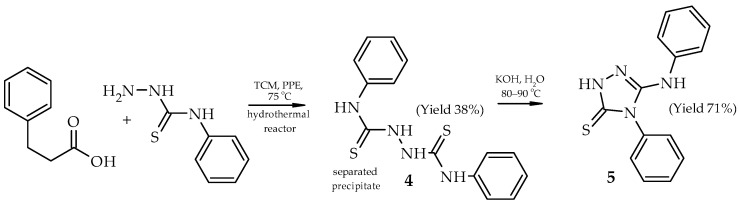
Attempted synthesis of **2n** by reducing the amount of PPE, reaction time, and temperature.

Despite the fairly diverse set of carboxylic acids used (Table 3), the obtained data are somewhat contradictory, making it difficult to identify any unambiguous correlation between the structure of the carboxylic acid and the yield of the target product. One possible reason for this variation in yields could be the difference in solubility of the acylation products in the reaction mixture: if the solubility of intermediate **1** is low, it precipitates at the first stage without undergoing further cyclodehydration to the thiadiazole. Based on this, it can be assumed that modifying the conditions of the “hydrothermal” stage could influence the yield of the target compounds. To test this hypothesis, we conducted additional attempts to synthesize **2n** and **2o** by varying the conditions of the first stage. In a new attempt to synthesize **2n**, the reaction time, amount of chloroform, and PPE were significantly reduced. This indeed led to the formation of a precipitate (analogous to syntheses **2a–h**). However, upon investigating the structure of the isolated product (and the product of its subsequent transformation under alkaline conditions), it was found that under the new conditions, the reaction proceeds according to Scheme 3, yielding products **4** and **5**.

It is worth noting that the mechanism of formation of product **4** is unlikely to be related to the presence of PPE, as a similar conversion of phenylthiosemicarbazide occurs without dehydration (furthermore, the formation of **4** was observed when the amount of PPE was reduced). It is more probable that the observed effect is associated with the specific nature of 4-phenylthiosemicarbazide, which under certain conditions can convert into compound **4** [68]. Thus, it is evident that the reaction with 4-phenylthiosemicarbazide can be further complicated by the potential formation of product 4, which may ultimately affect the yield of the target compounds **2**.

**Scheme 4:**
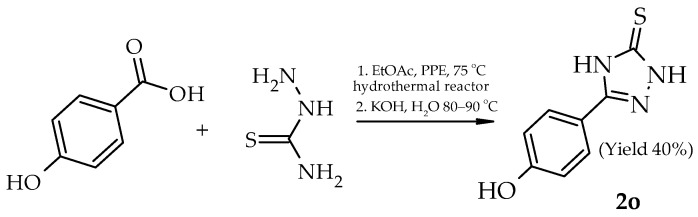
Synthesis of **2o** via solvent replacement (chloroform with ethyl acetate).

An attempt to carry out the synthesis of **2o** under the conditions of Scheme 3 did not yield any significant results (TLC analysis showed that after 4 h, the reaction mixture contained **3o** and the starting materials). A fundamentally different outcome was achieved by replacing chloroform with ethyl acetate as the solvent (Scheme 4). The successful implementation of Scheme 4 and the isolation of **2o** provide indirect confirmation of the hypothesis regarding the influence of the acylation product’s solubility in the reaction mixture. However, applying ethyl acetate to the synthesis of **2n** resulted in a homogeneous and difficult-to-separate reaction mixture, demonstrating the need to optimize the solvent for each specific structure and further indirectly confirming the importance of the solubility factor for a successful synthesis.

**Scheme 5:**
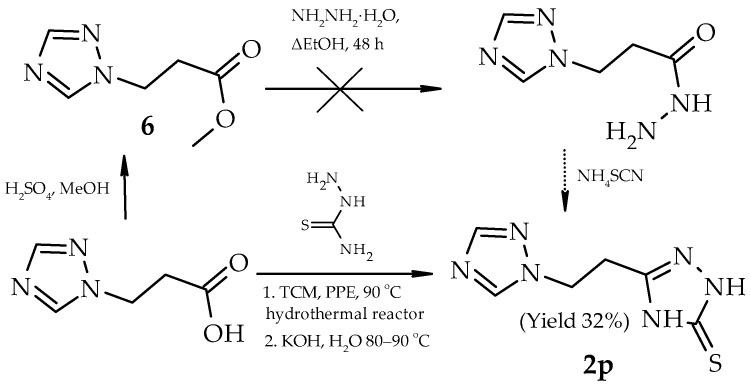
Comparative synthesis of **2p**: developed method vs. hydrazide/ammonium thiocyanate route.

The practical significance of the proposed synthetic method for 1,2,4-triazole-3-thiols becomes evident in cases where the synthesis of the requisite hydrazides for the classical isothiocyanate-based approach is challenging or requires specific, non-standard conditions. A prime example of this problem is the synthesis of compound **2p** (Scheme 5) starting from triazolyl propanoic acid.

We found that the reaction of methyl ester **6** with hydrazine in ethanol (Scheme 5) or isopropanol (detailed procedures are provided in the Supplementary Materials) does not yield the target hydrazide, even after 48 h. It is noteworthy that a structurally similar compound, 3-(1*H*-benzotriazol-1-yl)propanehydrazide, forms without any issues under the conditions shown in Scheme 5 [69]. The application of thionyl chloride, according to method [63], for the synthesis of **2p** was also unsuccessful, as the formation of a complex, difficult-to-separate mixture of by-products made the isolation of the target compound virtually impossible. In contrast, the reaction of triazolyl propanoic acid with thiosemicarbazide under the conditions developed for **2i–m** allowed for the straightforward isolation of compound **2p** in pure form (Scheme 5). It is important to emphasize that the aforementioned experiment does not prove the absolute impossibility of synthesizing the hydrazide or the acid chloride of 3-(1*H*-1,2,4-triazol-1-yl)propanoic acid. Rather, it indicates that finding the optimal conditions for these intermediates would require a separate investigation, distinct from classical approaches. Therefore, under the current circumstances, the method described in this work represents the optimal pathway for the laboratory synthesis of compound **2p**.

## 3. Materials and Methods

Starting materials. Commercial reagents (purity ≥ 97%) from Sigma-Aldrich (Steinheim, Germany) and Alladin Scientific (Shanghai, China) were used as starting materials. 1*H*-Benzotriazole-1-propanoic acid was prepared according to the procedure reported in [70]. 3-(1*H*-1,2,4-triazol-1-yl)propanoic acid was prepared according to the procedure reported in [71]. Polyphosphate ester was synthesized as described in [72].

Equipment. The following equipment was used for analysis: a Finnigan MAT INCOS 50 mass spectrometer (Finnigan Instrument Corporation, Bremen, Germany), a Bruker DRX-500 spectrometer (Bruker, Karlsruhe, Germany), and a Vario MICRO cube CHNS analyzer (Elementar Analysensysteme GmbH, Langenselbold, Germany). Synthesis of compounds **2** and **3** was performed using a 10 mL hydrothermal reaction vessel (PTFE with a stainless-steel jacket, Xi’an Taikang Biotechnology Co., Ltd., Xi’an, China).

General procedure for the synthesis of **1a–c**. Benzoic acid (8.2 mmol) and thiosemicarbazide (8.2 mmol) were thoroughly mixed with a spatula in a 10 mL vial. Chloroform (6 mL) was added to the mixture, and the suspension was stirred at room temperature for 5 min. Subsequently, PPE (1.5 g) was added to the reaction mixture, which was stirred at 64 °C for 3 h and then cooled to room temperature. Precipitation occurred within 24 h. The resulting precipitate was filtered off, washed with chloroform and a water (90%)/methanol (10%) mixture.

General procedure for the synthesis of **2a–m**. A carboxylic acid (8.2 mmol) and thiosemicarbazide (8.2 mmol) were thoroughly mixed with a spatula in a 10 mL hydrothermal reaction vessel. Dry chloroform (4 mL) and a stir bar were added to the mixture. The open vessel was placed on a magnetic stirrer. Then, PPE (1.5 g) was added to the reaction mixture under stirring, the vessel was sealed, and the jacket temperature was maintained at 90 °C for 11 h.

Isolation of **2a–h**. The formed precipitate was filtered off, washed with chloroform, and suspended in water (15 mL). A 2 M KOH solution was added to adjust the pH to 9–10. The resulting mixture was stirred at 90 °C for 9 h, maintaining the pH at ~9–10 by periodic addition of 2 M KOH. Upon completion, any undissolved precipitate (except for **2e**) was discarded. The filtrate was treated with activated charcoal (~80 mg) and acidified with 0.5 M HCl to pH ~2. The resulting precipitate was filtered off and washed with a water (90%)/methanol (10%) mixture. In the case of **2e**, after 9 h of alkaline treatment, no significant dissolution of the precipitate was observed. Therefore, the precipitate was filtered off, dissolved in methanol (40 mL), and acidified with 0.5 M HCl to pH ~2. The formed precipitate was filtered off and washed with a water (90%)/methanol (10%) mixture.

Isolation of **2i–m**. The primary difference from the procedure for **2a–h** is that the reaction in the presence of PPE yields a resin-like mass instead of a precipitate. This mass was separated from chloroform by decantation, transferred into water (15 mL), and the subsequent steps were identical to those described for **2a–h**.

Isolation of **3n** from the reaction mixture targeting compound **2n**. The resulting mixture was evaporated. The residue was mixed with water (15 mL), and a 2 M KOH solution was added to adjust the pH to 9–10. The mixture was stirred at 70 °C for 1 h. The formed precipitate was filtered off and washed with isopropyl alcohol (2 × 15 mL) and methanol (2 × 15 mL).

Isolation of **3o** from the reaction mixture targeting compound **2o**. The formed resin-like mass was separated from the chloroform residue and transferred into water (15 mL). A 2 M KOH solution was added to adjust the pH to 9–10, and the mixture was stirred at 80 °C for 2 h. Subsequently, the pH was adjusted to 6 by adding 0.5 M HCl. The precipitate was filtered off and washed with a water (90%)/methanol (10%) mixture.

Synthesis of **4** by the reaction targeting compound **2n**. Phenylpropanoic acid (1.23 g) and 4-phenylthiosemicarbazide (1.37 g) were thoroughly mixed with a spatula in a 10 mL hydrothermal reaction vessel. Dry chloroform (2 mL) and a stir bar were added to the mixture. The open vessel was placed on a magnetic stirrer. Then, PPE (0.85 g) was added to the reaction mixture under stirring, the vessel was sealed, and the jacket temperature was maintained at 75 °C for 4 h. The formed precipitate was filtered off and washed with chloroform. Yield: 0.48 g (38%, based on the stoichiometry of formation of **4**).

Synthesis of **5**. A mixture of *N*^1^,*N*^2^-diphenylhydrazine-1,2-dicarbothioamide **4** (0.3 g) and KOH (0.15 g) in water (15 mL) was stirred at 90 °C for 4 h. The reaction mixture was then cooled to room temperature and acidified with HCl to pH ~6. The resulting precipitate was filtered off, washed with water and a water (90%)/methanol (10%) mixture. Yield: 0.19 g (71%).

Synthesis of **2o**. 4-Hydroxybenzoic acid (1.13 g) and thiosemicarbazide (0.75 g) were thoroughly mixed with a spatula in a 10 mL hydrothermal reaction vessel. Dry ethyl acetate (2 mL) and a stir bar were added to the mixture. The open vessel was placed on a magnetic stirrer. Then, PPE (0.85 g) was added to the reaction mixture under stirring, the vessel was sealed, and the jacket temperature was maintained at 75 °C for 4 h. The resulting reaction mixture was transferred into ethyl acetate (50 mL) and treated in an ultrasonic bath for 30 min, yielding three fractions: an ethyl acetate solution, a colorless precipitate, and a resin-like mass (the resin was separated by decantation and discarded). The obtained precipitate was filtered off, washed with ethyl acetate, and treated with an aqueous alkali solution according to the procedure described above (see “Isolation of **2a–h**”). Yield: 0.64 g (40%).

Synthesis of **2p**. The synthesis and isolation of the product were carried out according to the general procedure described for compounds **2i–m**, with a single modification: the resulting precipitate was additionally washed with acetone (3 × 20 mL).

Compounds **2e**, **2k**, **2l**, **2m** and **2p** were obtained for the first time. The following compounds were described previously: **1a** [73], **1b** [74], **1c** [75], **2a** [27], **2b** [74], **2c** [76], **2d** [77], **2f** [78], **2g** [79], **2h** [80], **2i** [81], **2j** [82], **2o** [74], **3n** [83], **3o** [84], **4** [68], **5** [85], **6** [86]. Spectral data are provided in the Supplementary Materials.

## 4. Conclusions

For the first time, the possibility of synthesizing 1,2,4-triazole-3-thiol derivatives via a direct reaction of thiosemicarbazides with carboxylic acids using polyphosphate ester (PPE) has been demonstrated. The synthesis is carried out in two stages: (i) acylation of the thiosemicarbazide with carboxylic acid in chloroform in the presence of PPE, followed by (ii) cyclodehydration of the resulting acylation product by treatment with an aqueous alkali solution. It was shown that the use of a hydrothermal reaction vessel is optimal for the acylation stage, as the simultaneous increase in temperature and pressure simplifies the isolation of the intermediate acylation product for the second synthesis stage. The found conditions for the acylation stage can be considered nominally optimal; however, it was demonstrated that in some cases, the process can proceed further to form thiadiazoles. This effect is indirectly shown to be primarily associated with the solubility of the acylation product in the reaction mixture: to halt the reaction at the acylation stage and avoid thiadiazole formation, a solvent system most conducive to the precipitation of the acylation product must be selected (i.e., optimal conditions must be determined individually for each reaction to maximize the target product yield). Furthermore, it was shown that the use of a hydrothermal reaction vessel significantly reduces the amount of PPE required for the synthesis of thiadiazoles compared to previously reported procedures [67]. Additionally, it was demonstrated that PPE can be used for the acylation of thiosemicarbazides with 4-hydroxybenzoic acid without prior protection of the hydroxyl group, unlike the method described, for example, in [84]. Thus, the combination of PPE and a hydrothermal reactor has been shown to be highly effective for conducting dehydration and cyclodehydration reactions, a strategy that could find application not only in the synthesis of triazoles or thiadiazoles but also in the synthesis of other heterocycles via reactions involving hydrazides, amides, diacyl hydrazines, amino acids, etc.

## Figures and Tables

**Table 1:** Therapeutic targets of 1,2,4-triazole-3-thiol derivatives.

Therapeutic Areas	Targets		
Cancer	ATPase VCP/p97 [5]	DHFR [13], FUT6 [14]	PARP-1 [22,23]
	BRD4 [6]	HDACs [15,16]	Tdp1 [24]
	CA I, II, IV and IX [7,8,9,10]	Hsp90 [17,18]	TNKS1/2 [25]
	CK1γ3 [11]	KDM1A [19]	PARP-5b [26]
	DCN1-UBC12 int. [12]	MMPs [7,20,21]	TYMP [27]
Diabetes	α-AMY [28], AGase [28,29,30,31]	11b-HSD1 [32]	PTP1B [33]
Inflammatory disorders	ALOX15 [34,35]	COX1 [36,37]	ELANE [38]
Neurological disorders	AChE [30,39,40,41,42,43,44]	AChRε [45]	MPO [47]
	BChE [30,40,41,42,43,44]	mGlu5 [46]	PKC [48]
Respiratory disorders	NA [49], PDE4B [50]	PDE10A [51]	RNA pol. [35]
Bacterial infections	MBLs [52,53,54,55,56]	METAP1 [57]	Urease [58]

**Table 2:** Antimicrobial activity of 1,2,4-triazole-3-thiol derivatives.

Activity Against	Strains	
Gram-positive bacteria	*A. oxydans* [59]	*M. smegmatis* [59]
	*B. cereus* [59]	*M. tuberculosis* [63,64,65]
	*B. subtilis* [13,60,61,62]	*S. aureus.* [13,59,60,61,62,66]
	*E. faecalis* [59]	*S. pneumoniae* [60]
Gram-negative bacteria	*Acinetobacter* sp. [59]	*P. aeruginosa* [13,59,60,61]
	*E. coli* [13,59,60,61,62,66]	*P. vulgaris* [59]
	*K. oxytoca* [59]	*S. marcescens* [59]
	*K. pneumoniae* [60,66]	YPTB [59]
Fungi	*A. flavus* [66]	*M. anisopliae* [62]
	*A. fumigatus* [60,66]	*P. marneffei* [66]
	*A. niger* [61,62]	*S. cerevisiae* [59]
	*C. albicans* [13,59,60,61]	*T. harzianum* [62]
	*C. tropicalis* [59]	*T. mentagrophytes* [66]

**Table 3:** Main results of the experimental implementation of Scheme 2.

Compound	R^1^	R^2^	Yield, %
**2a**	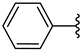	H	30
**2b**		Ethyl	27
**2c**		Phenyl	22
**2d**	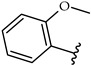	H	34
**2e**	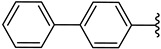	H	15
**2f**	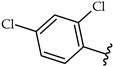	H	34
**2g**	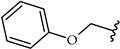	H	54
**2h**	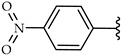	Ethyl	20
**2i**	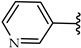	H	37
**2j**		Ethyl	29
**2k**	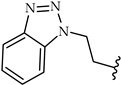	H	35
**2l**		Ethyl	13
**2m**		Phenyl	35
**2n**	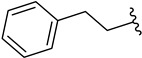	Phenyl	-
**2o**	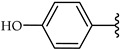	H	-

## Data Availability

The authors confirm that the data supporting the findings of this study are available within the article and its Supplementary Materials.

## Supplementary Materials

The following supporting information can be downloaded at https://www.mdpi.com/article/10.3390/molecules30224422/s1. Description of spectral data, and NMR and mass spectra images of the obtained compounds (Figures S1–S37), along with an additional synthesis procedure for compound **6** and a description of attempts to carry out the reaction of **6** with hydrazine hydrate.
