# Supplementary material for: Synthesis of 1,2,4-Triazole-3-Thiol Derivatives from Thiosemicarbazides and Carboxylic Acids Using Polyphosphate Ester
Source: Molecules. 2025 Nov 16;30(22):4422. doi: 10.3390/molecules30224422 (PMC12655543; doi:10.3390/molecules30224422)
Supplement: Supplementary file 1 [file molecules-30-04422-s001.zip › molecules-3934631-supplementary.pdf]

Synthesis of 1,2,4-Triazole-3-thiol Derivatives from Thiosemicarbazides and Carboxylic Acids Using Polyphosphate Ester

Bogdan A. Tretyakov [1], Viktoria I. Tikhonova [1,2], Svyatoslav Y. Gadomsky [1,*] and Nataliya A. Sanina [1]

2-benzoylhydrazine-1-carbothioamide (1a). Yield 0.56 g (35%). ^{1}H NMR spectrum (DMSO-d_6), ppm (*J*, Hz): 7.47 (2H, t, *J* = 7.6, H Ar), 7.56 (1H, t, *J* = 7.3, H Ar), 7.62 (1H, s, -NH_2), 7.86 (1H, s, -NH_2), 7.90 (2H, d, *J* = 7.4, H Ar), 9.33 (1H, s, -NH-), 10.34 (1H, s, -NH-).

2-benzoyl-*N*-ethylhydrazine-1-carbothioamide (1b). Yield 0.47 g (26%). ^{1}H NMR spectrum (DMSO-d_6), ppm (*J*, Hz): 1.06 (3H, t, *J* = 7.1, -CH_3), 3.47 (2H, dt, J_1 = 6.8, J_2 = 13.2, -CH_2-) 7.49 (2H, t, *J* = 7.7, H Ar), 7.58 (1H, t, *J* = 7.3, H Ar), 7.89-7.94 (2H, m, H Ar), 8.09 (1H, s, -NH-), 9.24 (1H, s, -NH-), 10.30 (1H, s, -NH-).

2-benzoyl-*N*-phenylhydrazine-1-carbothioamide (1c). Yield 0.36 g (16%). ^{1}H NMR spectrum (DMSO-d_6), ppm (*J*, Hz): 7.15 (1H, t, *J* = 7.3, H Ar), 7.33 (2H, t, *J* = 7.8, H Ar), 7.39-7.53 (4H, m, H Ar), 7.58 (1H, t, *J* = 7.3, H Ar), 7.96 (2H, d, *J* = 7.5, H Ar), 9.70 (1H, s, -NH-), 9.81 (1H, s, -NH-), 10.53 (1H, s, -NH-).

5-phenyl-2,4-dihydro-3H-1,2,4-triazole-3-thione (2a). Yield 0.44 g (30%). ^{1}H NMR spectrum (DMSO-d_6), ppm (*J*, Hz): 7.49-7.55 (3H, m, H Ar), 7.90-7.93 (2H, m, H Ar), 13.40-14.15 (2H, bs, -NH-, -SH).

4-ethyl-5-phenyl-2,4-dihydro-3*H*-1,2,4-triazole-3-thione (2b). Yield 0.45 g (27%). ^{1}H NMR spectrum (DMSO-d_6), ppm (*J*, Hz): 1.15 (3H, t, *J* = 7.1, -CH_3), 4.04 (2H, q, *J* = 7.1, -CH_2-), 7.57-7.64 (3H, m, H Ar), 7.68 (2H, d, *J* = 6.6, H Ar), 13.92 (1H, s, -NH-, -SH).

4,5-diphenyl-2,4-dihydro-3*H*-1,2,4-triazole-3-thione (2c). Yield 0.46 g (22%). ^{1}H NMR spectrum (DMSO-d_6), ppm (*J*, Hz): 7.28-7.37 (6H, m, H Ar), 7.41 (1H, t, *J* = 7.1, H Ar), 7.47-7.52 (3H, m, H Ar), 14.13 (1H, s, -NH-, -SH).

5-(2-methoxyphenyl)-2,4-dihydro-3H-1,2,4-triazole-3-thione (2d). Yield 0.58 g (34%). ^{1}H NMR spectrum (DMSO-d_6), ppm (*J*, Hz): 3.86 (3H, s, O-CH_3), 7.06 (1H, t, *J* =7.5, H Ar), 7.18 (1H, d, *J* = 8.4, H Ar), 7.51 (1H, t, *J* = 7.9, H Ar), 7.65 (1H, d, *J* = 7.6, H Ar), 12.9-13.9 (2H, m, -NH-, -SH).

5-([1,1'-biphenyl]-4-yl)-2,4-dihydro-3*H*-1,2,4-triazole-3-thione (2e). Yield 0.31 g (15%). Found, %: C 66.35; H 4.41; N 16.64; S 12.59. $C_{14}H_{11}N_3S$. Calculated, %: C 66.38; H 4.38; N 16.59; S 12.66. Mass spectrum, m/z (I_{rel}, %): 254 $[M]^+$ (100), 194 (14), 180 (17), 152 (11). ^{1}H NMR spectrum (DMSO-d_6), ppm (*J*, Hz): 7.42 (1H, t, *J* = 7.2, H Ar), 7.51 (2H, t, *J* = 7.5, H Ar), 7.75 (2H, d, *J* = 7.5, H Ar), 7.84 (2H, d, *J* = 8.2, H Ar), 8.02 (2H, d, *J* = 8.2, H Ar). 13.75 (1H, s, -NH-, -SH), 13.93 (1H, s, -NH-, -SH). ^{13}C NMR spectrum (DMSO-d_6), δ, ppm: 124.8; 126.7; 127.2; 127.7; 128.5; 129.5; 139.4; 142.5; 150.4; 167.5.

5-(2,4-dichlorophenyl)-2,4-dihydro-3H-1,2,4-triazole-3-thione (2f). Yield 0.69 g (34%). ^{1}H NMR spectrum (DMSO-d_6), ppm (*J*, Hz): 7.60 (1H, d, *J* = 8.4, H Ar), 7.71 (1H, d, *J* = 8.4, H Ar), 7.84 (1H, s, H Ar), 13.55-13.95 (2H, m, -NH-, -SH).

5-(phenoxymethyl)-2,4-dihydro-3*H*-1,2,4-triazole-3-thione (2g). Yield 0.91 g (54%). ^{1}H NMR spectrum (DMSO-d_6), ppm (*J*, Hz): 5.02 (2H, s, - CH_2-), 6.95-7.06 (3H, m, H Ar), 7.28-7.34 (2H, m, H Ar), 13.2-14.3 (2H, m, -NH-, -SH).

4-ethyl-5-(4-nitrophenyl)-2,4-dihydro-3*H*-1,2,4-triazole-3-thione (2h). Yield 0.40 g (19.5%). ^{1}H NMR spectrum (DMSO-d_6), ppm (*J*, Hz): 1.18 (3H, t, *J* = 7.1, -CH_3), 1.18 (2H, q, *J* = 7.1, -CH_2-), 8.01 (2H, d, *J* = 8.6, H Ar), 8.40 (2H, d, *J* = 8.7, H Ar), 14.14 (1H, s, -NH-, -SH).

5-(pyridin-3-yl)-2,4-dihydro-3*H*-1,2,4-triazole-3-thione (2i). Yield 0.53 g (37%). ^{1}H NMR spectrum (DMSO-d_6), ppm (*J*, Hz): 7.53-7.58 (1H, m, H Ar), 8.22-8.26 (1H, m, H Ar), 8.67-8.70 (1H, m, H Ar), 9.05-9.08 (1H, m, H Ar), 13.82 (1H, s, -NH), 14.01 (1H, s, -NH-, -SH).

4-ethyl-5-(pyridin-3-yl)-2,4-dihydro-3*H*-1,2,4-triazole-3-thione (2j). Yield 0.49 g (29%). ^{1}H NMR spectrum (DMSO-d_6), ppm (*J*, Hz): 1.16 (3H, t, *J* = 7.2, -CH_3), 4.05 (2H, q, *J* = 7.2, -CH_2-), 7.62 (1H,

dd, J_1 = 7.8, J_2 = 4.9, H Ar), 8.15 (1H, dt, J_1 = 7.9, J_2 = 1.9, H Ar), 8.80 (1H, dd, J_1 = 4.8, J_2 = 1.5, H Ar), 8.88 (1H, d, *J* = 1.8, H Ar), 14.06 (1H, s, -NH-, -SH).

5-[2-(1*H*-benzotriazol-1-yl)ethyl]-2,4-dihydro-3*H*-1,2,4-triazole-3-thione (2k). Yield 0.71 g (35%). Found, %: C 48.69; H 4.21; N 34.19; S 12.98. $C_{10}H_{10}N_6S$. Calculated, %: C 48.77; H 4.09; N 34.12; S 13.02. Mass spectrum, m/z (I_{rel}, %): 246 $[M]^+$ (100), 190 (5), 185 (11), 128 (47), 115 (15), 104 (19), 91 (22), 77 (41). ^{1}H NMR spectrum (DMSO-*d*$_6$), ppm (*J*, Hz): 3.25 (2H, t, *J* = 6.8, -CH_2-), 5.04 (2H, t, *J* = 6.8, -CH_2-), 7.38-7.45 (1H, m, H Ar), 7.56 (1H, t, *J* = 7.6, H Ar), 7.85 (1H, d, *J* = 8.4, H Ar), 8.03 (1H, d, *J* = 8.3, H Ar), 13.05-13.50 (2H, m, -NH-, -SH). ^{13}C NMR spectrum (DMSO-*d*$_6$), δ, ppm: 26.3; 45.1; 110.9; 119.6; 124.5; 127.8; 133.2; 145.5; 149.7; 166.6.

5-[2-(1*H*-benzotriazol-1-yl)ethyl]-4-ethyl-2,4-dihydro-3*H*-1,2,4-triazole-3-thione (2l). Yield 0.30 g (13%). Found, %: C 52.39; H 5.22; N 30.71; S 11.65. $C_{12}H_{14}N_6S$. Calculated, %: C 52.54; H 5.14; N 30.63; S 11.69. Mass spectrum, m/z (I_{rel}, %): 274 $[M]^+$ (100), 155 (65), 104 (20), 77 (44). ^{1}H NMR spectrum (DMSO-*d*$_6$), ppm (*J*, Hz): 1.16 (3H, t, *J* = 7.2, -CH_3), 3.46 (2H, t, *J* = 7.0, -CH_2-), 3.95 (2H, q, *J* = 7.1, -CH_2-), 5.09 (2H, t, *J* = 7.0, -CH_2-), 7.41 (1H, t, *J* = 7.6, H Ar), 7.56 (1H, t, *J* = 7.6, H Ar), 7.90 (1H, d, *J* = 8.3, H Ar), 8.04 (1H, d, *J* = 8.4, H Ar), 13.50 (1H, s, -NH-, -SH). ^{13}C NMR spectrum (DMSO-*d*$_6$), δ, ppm: 13.8; 25.6; 38.6; 44.5; 111.2; 119.6; 124.5; 127.7; 133.3; 145.6; 149.6; 166.6.

5-[2-(1*H*-benzotriazol-1-yl)ethyl]-4-phenyl-2,4-dihydro-3*H*-1,2,4-triazole-3-thione (2m). Yield 0.92 g (35%). Found, %: C 52.46; H 4.41; N 26.12; S 9.88. $C_{16}H_{14}N_6S$. Calculated, %: C 59.61; H 4.38; N 26.07; S 9.95. Mass spectrum, m/z (I_{rel}, %): 322 $[M]^+$ (85), 203 (100), 104 (24), 91 (24), 77 (83). ^{1}H NMR spectrum (DMSO-*d*$_6$), ppm (*J*, Hz): 3.16 (2H, t, *J* = 6.8, -CH_2-), 4.91 (2H, t, *J* = 6.9, -CH_2-), 7.32 (2H, d, *J* = 7.7, H Ar), 7.40 (1H, t, *J* = 7.5, H Ar), 7.48-7.59 (4H, m, H Ar), 7.72 (1H, d, *J* = 8.3, H Ar), 8.02 (1H, d, *J* = 8.3, H Ar), 13.75 (1H, s, -NH-, -SH). ^{13}C NMR spectrum (DMSO-*d*$_6$), δ, ppm: 26.3; 44.5; 110.8; 119.6; 124.5; 127.8; 128.6; 129.9; 130.0; 133.1; 133.7; 145.6; 149.7; 168.2.

5-(4-hydroxyphenyl)-2,4-dihydro-3H-1,2,4-triazole-3-thione (2o). Yield 0.64 g (40%). Mass spectrum, m/z (I_{rel}, %): 193 $[M]^+$ (100), 134 (22), 120 (26). ^{1}H NMR spectrum (DMSO-*d*$_6$), ppm (*J*, Hz): 6.87 (2H, d, *J* = 8.6, H Ar), 7.74 (2H, d, *J* = 8.6, H Ar), 10.06 (1H, s, -OH), 13,49 (1H, s, -NH, -SH), 13,60 (1H, s, -NH, -SH).

5-[2-(1H-1,2,4-triazol-1-yl)ethyl]-2,4-dihydro-3H-1,2,4-triazole-3-thione (2p). Yield 0.52 g (32%). Found, %: C 36.76; H 4.17; N 42.90; S 16.28. $C_6H_8N_6S$. Calculated, %: C 36.72; H 4.11; N 42.83; S 16.34. Mass spectrum, m/z (I_{rel}, %): 196 $[M]^+$ (60), 127 (100), 70 (15), 55 (30), 40 (29), 28 (38). ^{1}H NMR spectrum (DMSO-*d*$_6$), ppm (*J*, Hz): 3.10 (2H, t, *J* = 6.7, -CH_2-), 4.53 (2H, t, *J* = 6.7, -CH_2-) 7.95 (1H, s, CH=N), 8.47 (1H, s, CH=N), 13,05-13.35 (2H, m, -NH, -SH). ^{13}C NMR spectrum (DMSO-*d*$_6$), δ, ppm: 25.9; 45.4; 144.2; 149.3; 151.5; 166.1.

***N*-phenyl-5-(2-phenylethyl)-1,3,4-thiadiazol-2-amine (3n).** Yield 0.45 g (20%). Mass spectrum, m/z (I_{rel}, %): 281 $[M]^+$ (100), 190 (60), 136 (16), 91 (43), 77 (23), 65 (17). ^{1}H NMR spectrum (DMSO-*d*$_6$), ppm (*J*, Hz): 3.01 (2H, t, *J* = 7.6, -CH_2-), 3.24 (2H, t, *J* = 7.6, -CH_2-), 6.97 (1H, t, *J* = 7.3, H Ar), 7.17-7.24 (1H, m, H Ar), 7.25-7.35 (6H, m, H Ar), 7.58 (2H, d, *J* = 8.1, H Ar), 10.22 (1H, s, N-H).

4-(5-amino-1,3,4-thiadiazol-2-yl)phenol (3o). The compound is also described in [84]. Yield 0.7 g (44%). ^{1}H NMR spectrum (DMSO-*d*$_6$), ppm (*J*, Hz): 6.88 (2H, d, *J* = 7.7, H Ar), 7.07 (2H, s, -NH_2), 7.62 (2H, d, *J* = 7.6, H Ar), 10.06 (1H, s, -OH).

***N*^{1},*N*^{2}-diphenylhydrazine-1,2-dicarbothioamide (4)**. Mass spectrum, m/z (I_{rel}, %): 302 $[M]^+$ (28), 269 (100), 209 (23), 135 (48), 119 (32), 93 (77), 77 (97). ^{1}H NMR spectrum (DMSO-d6), ppm (*J*, Hz): 7.13 (2H, s, H Ar), 7.33 (4H, t, *J* = 7.4, H Ar), 7.55 (4H, d, *J* = 6.9), 9.3-10.15 (4H, m, N-H).

5-anilino-4-phenyl-2,4-dihydro-3*H*-1,2,4-triazole-3-thione (5). Mass spectrum, m/z (I_{rel}, %): 268 $[M]^+$ (100), 77 (39). ^{1}H NMR spectrum (DMSO-*d*$_6$), ppm (*J*, Hz): 6.91 (1H, t, *J* = 7.3, H Ar), 7.19-7.26 (2H, m, H Ar), 7.42 (4H, dd, J_1 = 12.8, J_2 = 4.8, H Ar), 7.51-7.60 (3H, m, H Ar), 8.33 (1H, s, -NH), 13.33 (1H, s, -NH-, -SH).

Methyl 3-(1*H*-1,2,4-triazol-1-yl)propanoate (6). ^{1}H NMR spectrum (DMSO-*d*$_6$), ppm (*J*, Hz): 2.90 (2H, t, *J* = 6.6, -CH_2-), 3.59 (3H, s, -CH_3), 4.42 (2H, t, J = 6.6, -CH_2-), 7.95 (1H, s, CH=N), 8.49 (1H, s, CH=N).

(1a) 2-benzoylhydrazine-1-carbothioamide

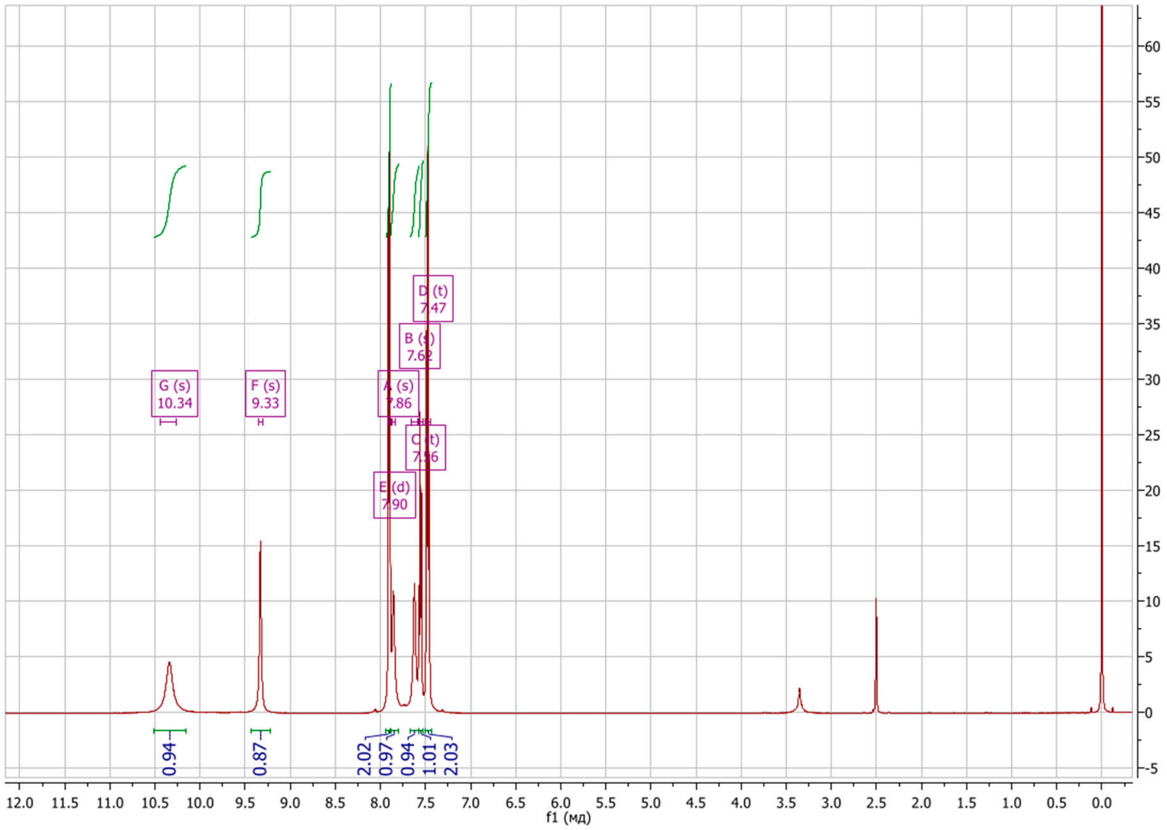

Figure S1. ^{1}H NMR spectrum of 1a (DMSO-d_6)

(1b) 2-benzoyl-*N*-ethylhydrazine-1-carbothioamide

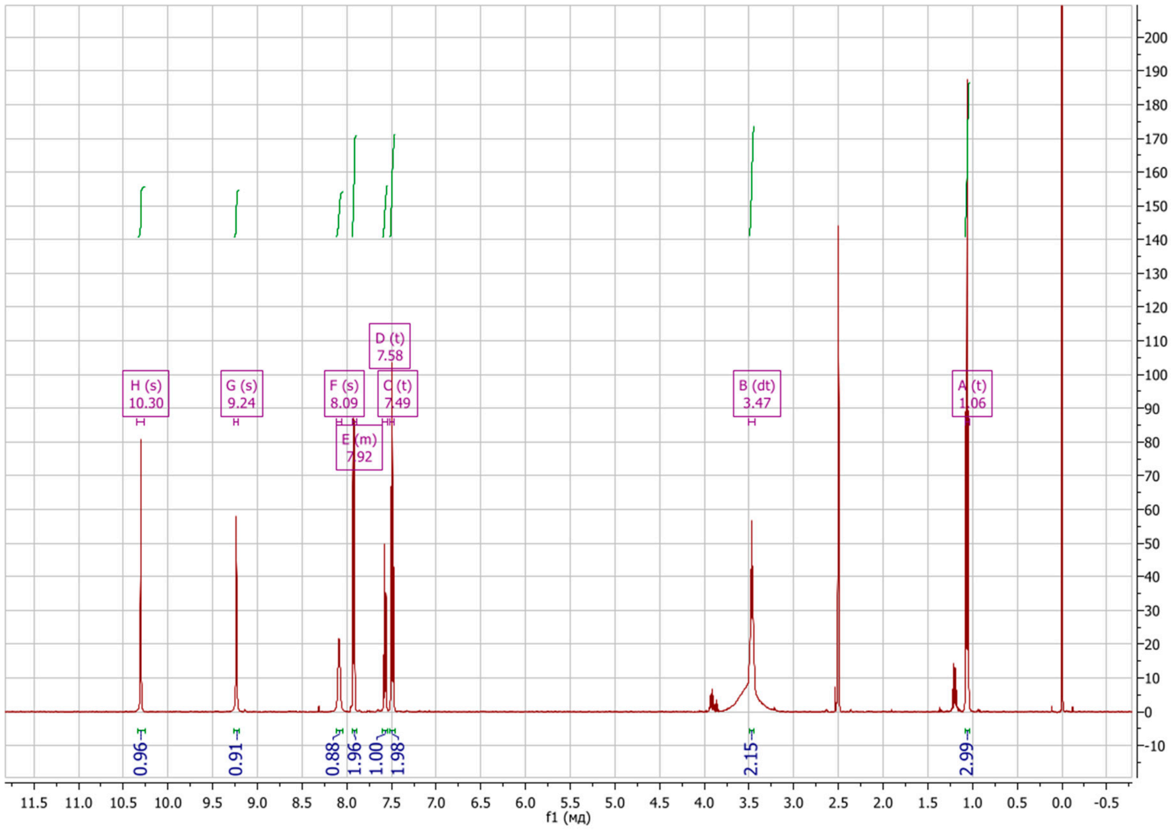

Figure S2. ^{1}H NMR spectrum of 1b (DMSO-*d*$_6$)

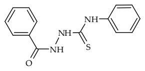

(1c) 2-benzoyl-*N*-phenylhydrazine-1-carbothioamide

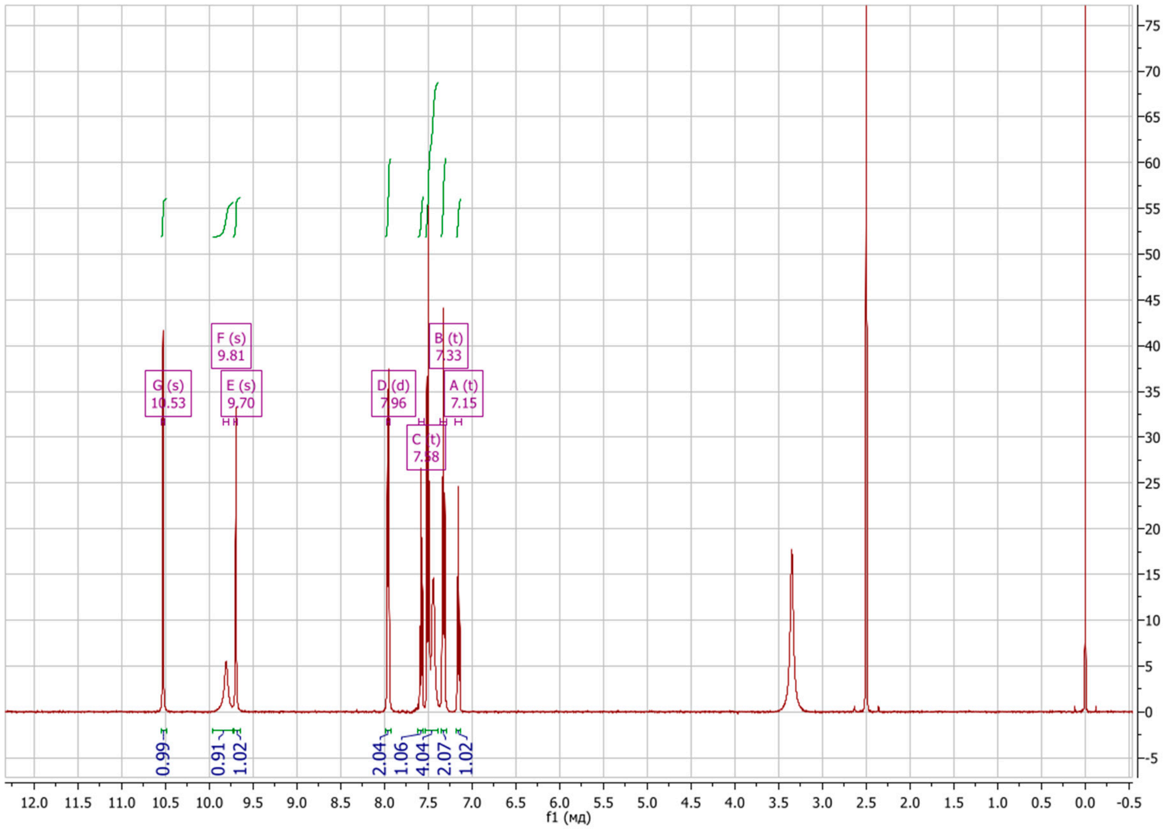

Figure S3. ^{1}H NMR spectrum of 1c (DMSO-d_6)

(2a) 5-phenyl-2,4-dihydro-3*H*-1,2,4-triazole-3-thione

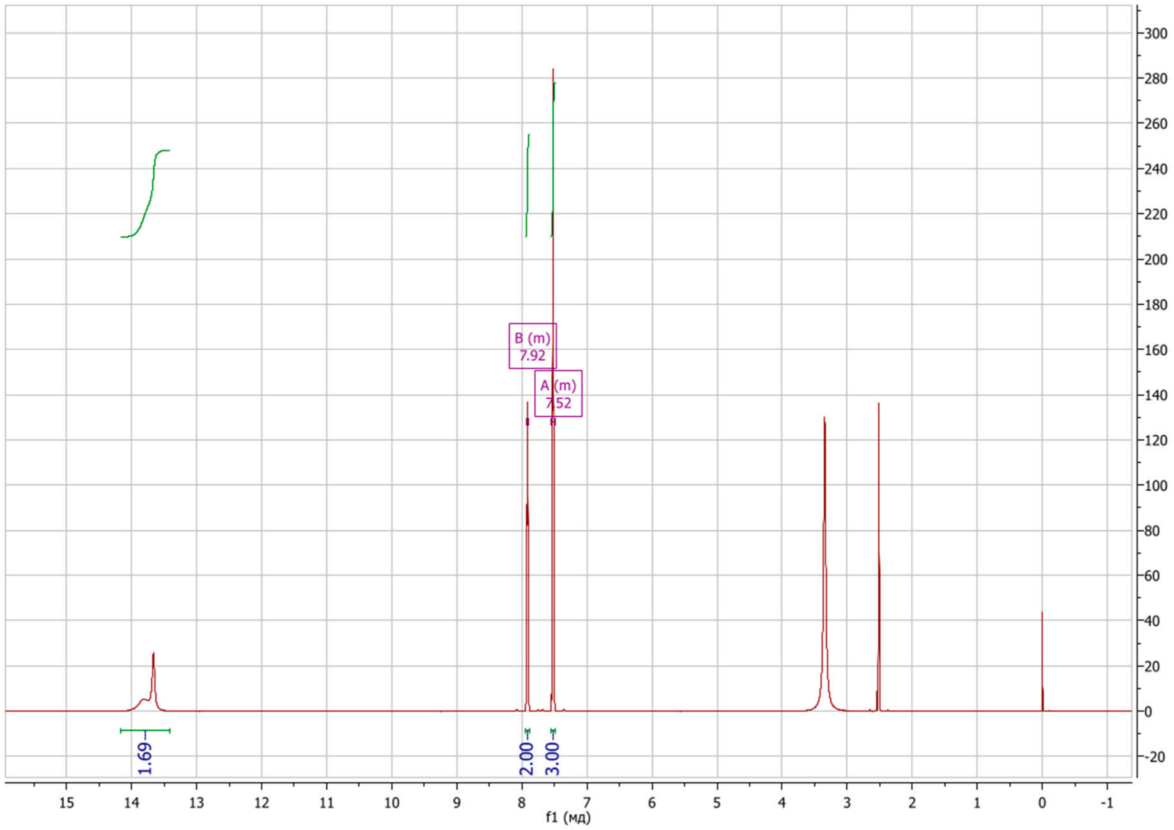

Figure S4. ^{1}H NMR spectrum of 2a (DMSO-d_6)

(2b) 4-ethyl-5-phenyl-2,4-dihydro-3*H*-1,2,4-triazole-3-thione

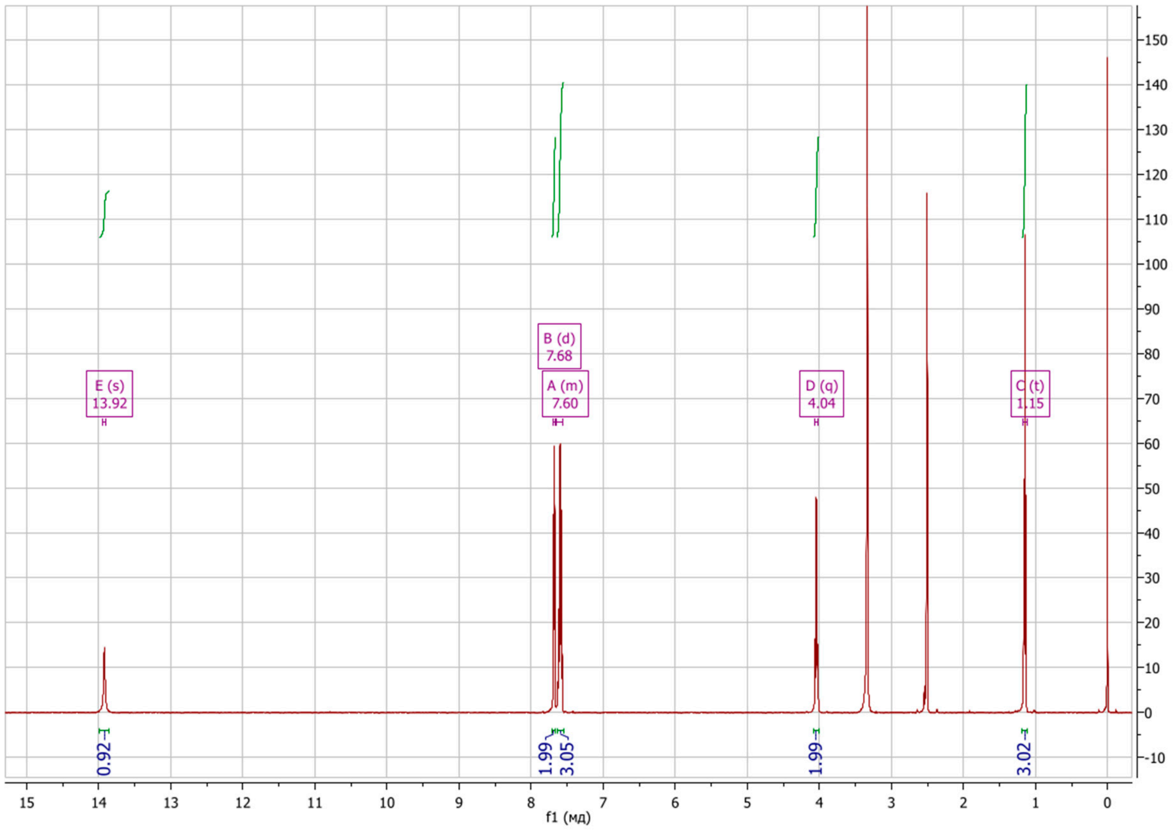

Figure S5. ^{1}H NMR spectrum of 2b (DMSO-d_6)

(2c) 4,5-diphenyl-2,4-dihydro-3*H*-1,2,4-triazole-3-thione

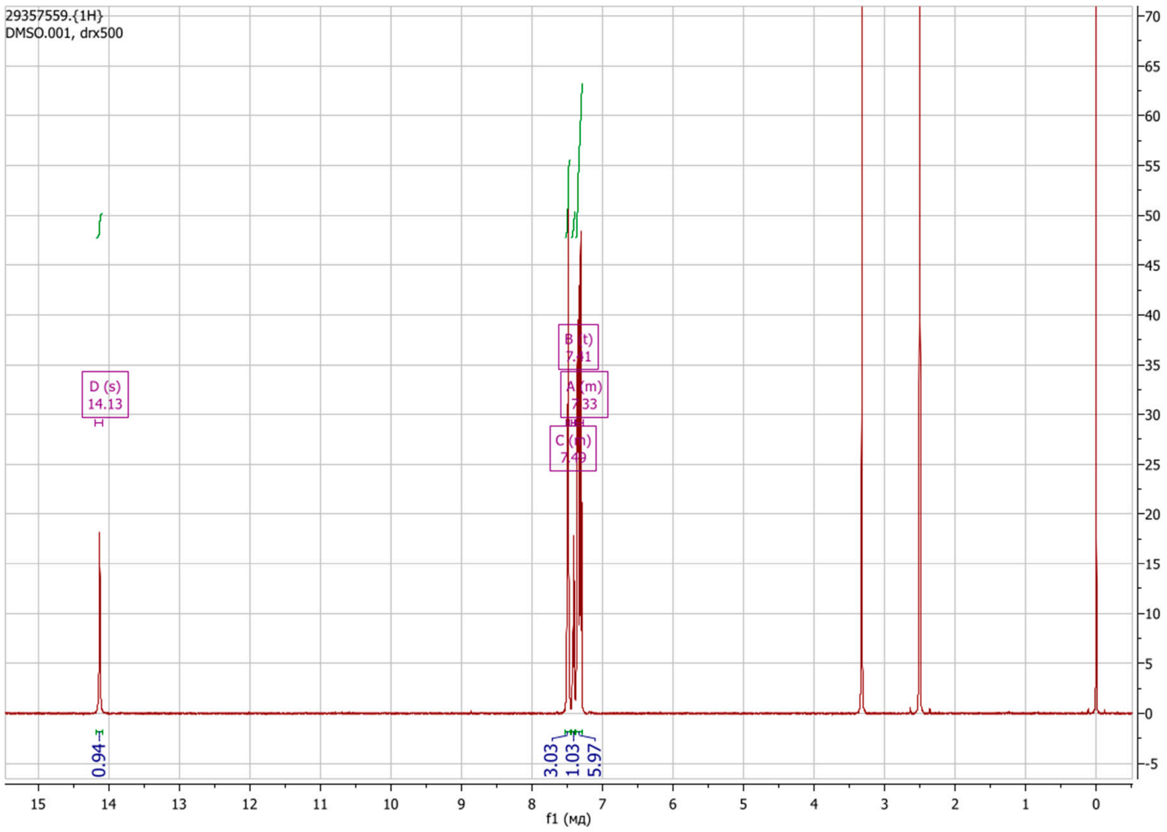

Figure S6. ^{1}H NMR spectrum of 2c (DMSO-d_6)

(2d) 5-(2-methoxyphenyl)-2,4-dihydro-3*H*-1,2,4-triazole-3-thione

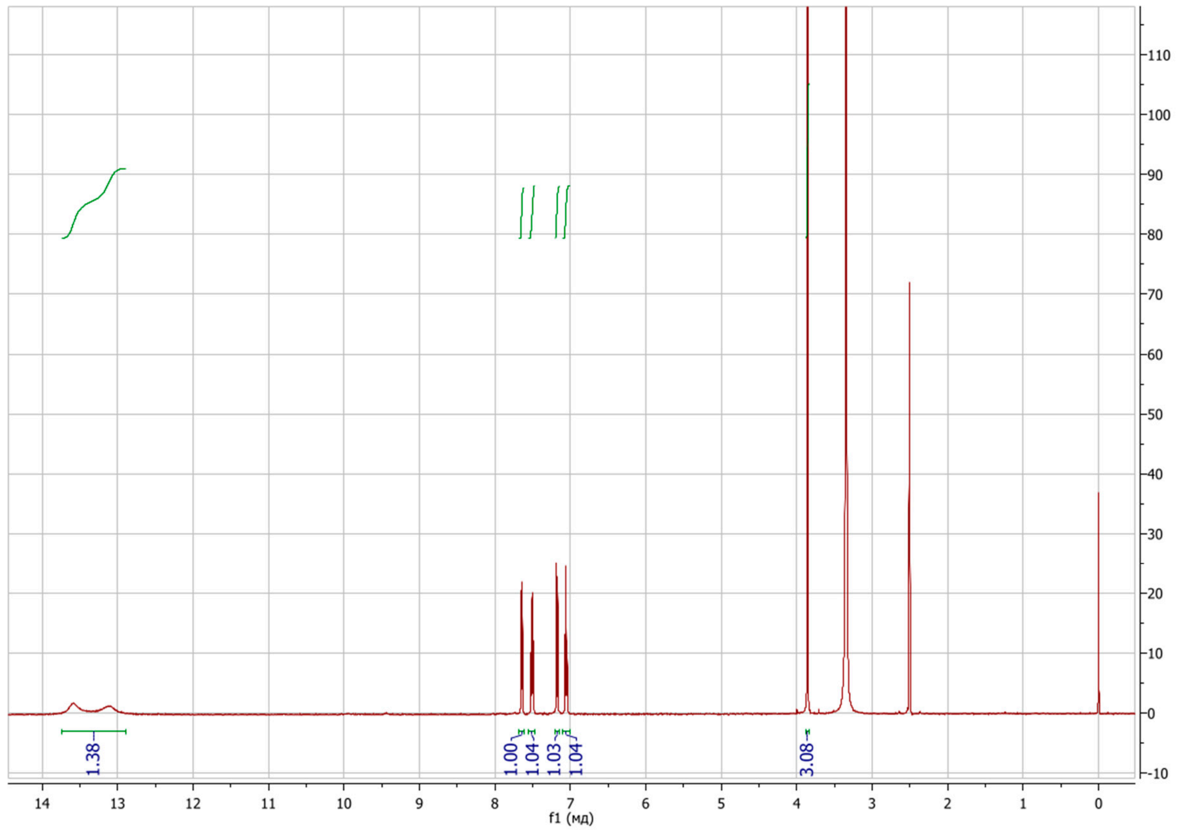

Figure S7. ^{1}H NMR spectrum of 2d (DMSO-d_6)

(2e) 5-([1,1'-biphenyl]-4-yl)-2,4-dihydro-3*H*-1,2,4-triazole-3-thione

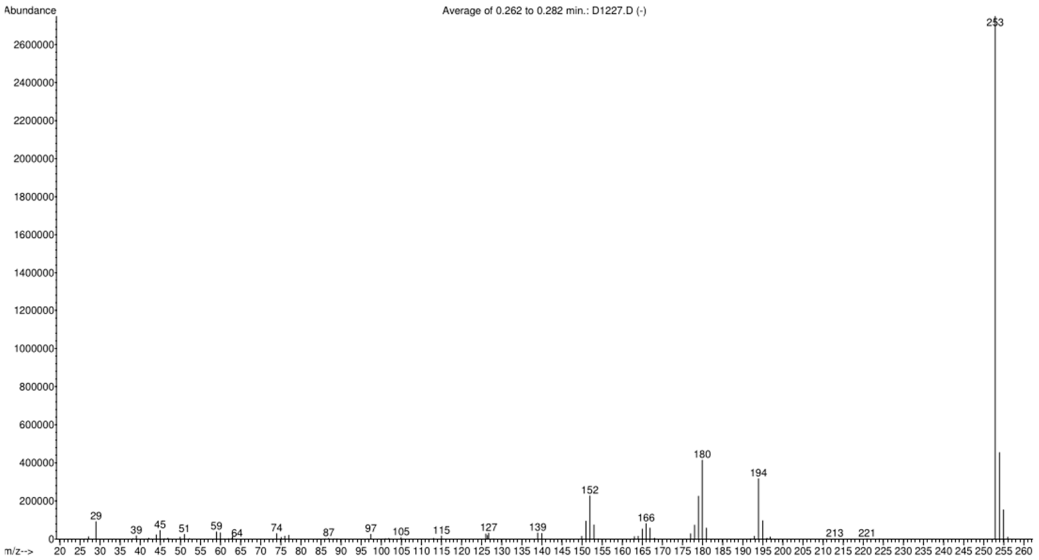

Figure S8. Mass spectrum of 2e

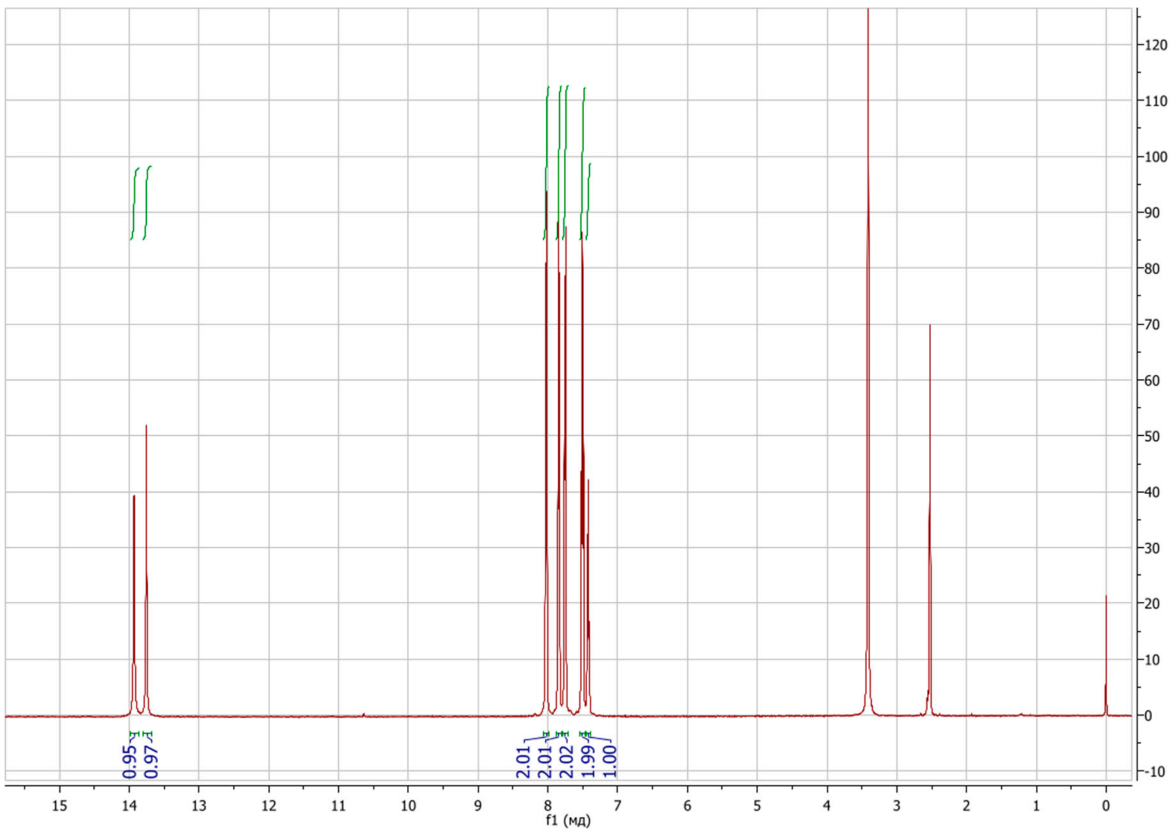

Figure S9. ^{1}H NMR spectrum of 2e (DMSO-d_6)

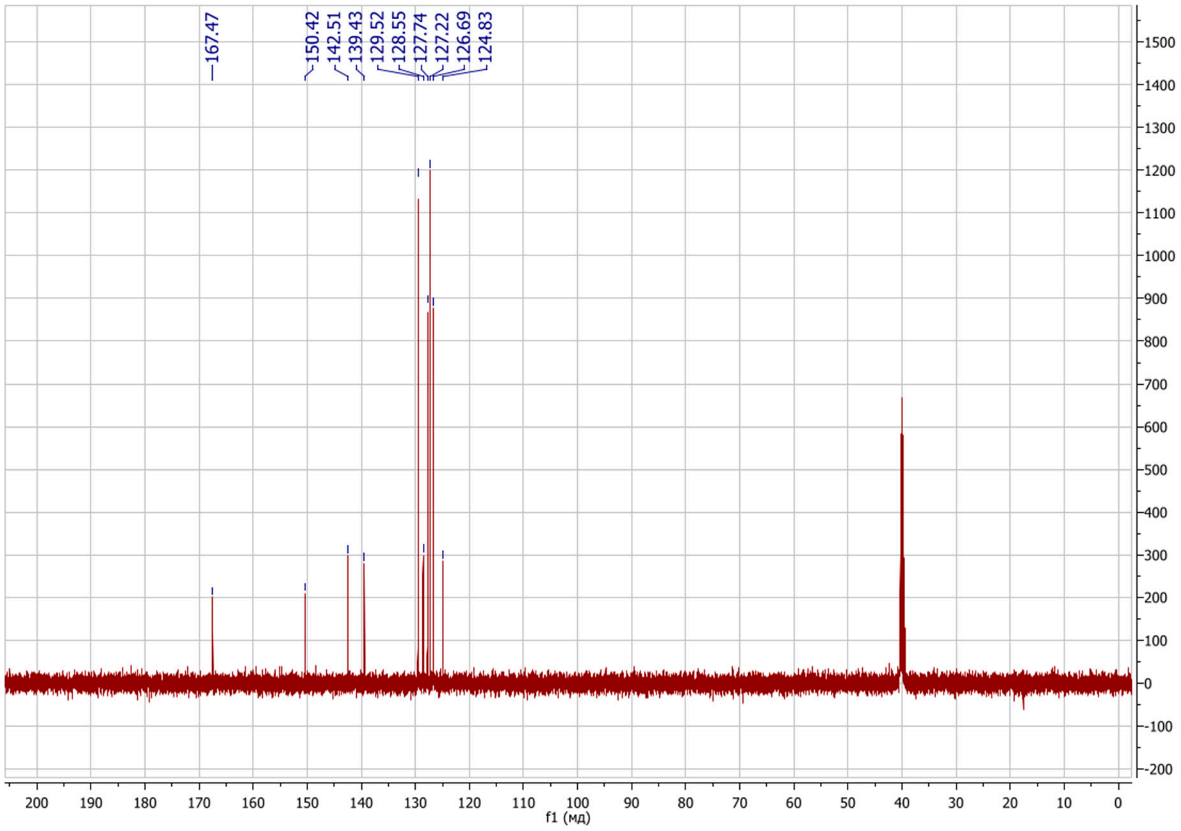

Figure S10. ^{13}C NMR spectrum of 2e (DMSO-d_6)

(2f) 5-(2,4-dichlorophenyl)-2,4-dihydro-3*H*-1,2,4-triazole-3-thione

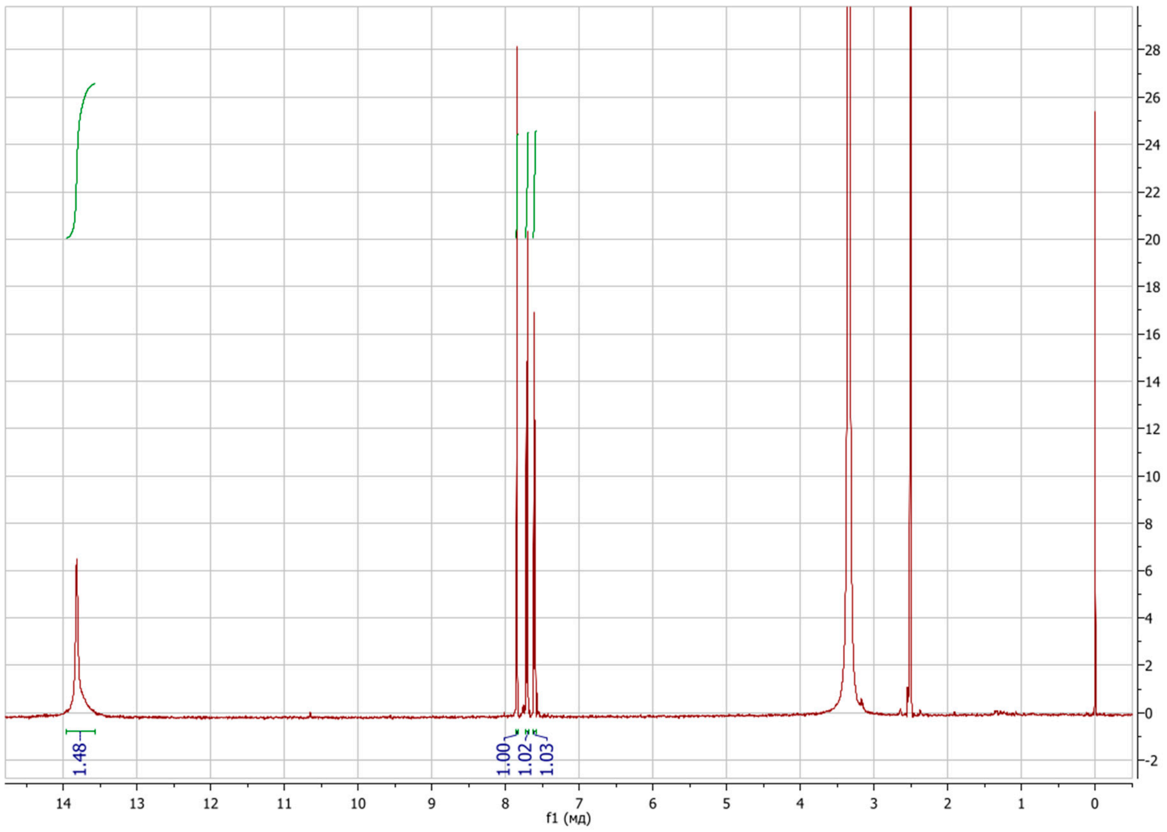

Figure S11. ^{1}H NMR spectrum of 2f (DMSO-d_6)

(2g) 5-(phenoxymethyl)-2,4-dihydro-3*H*-1,2,4-triazole-3-thione

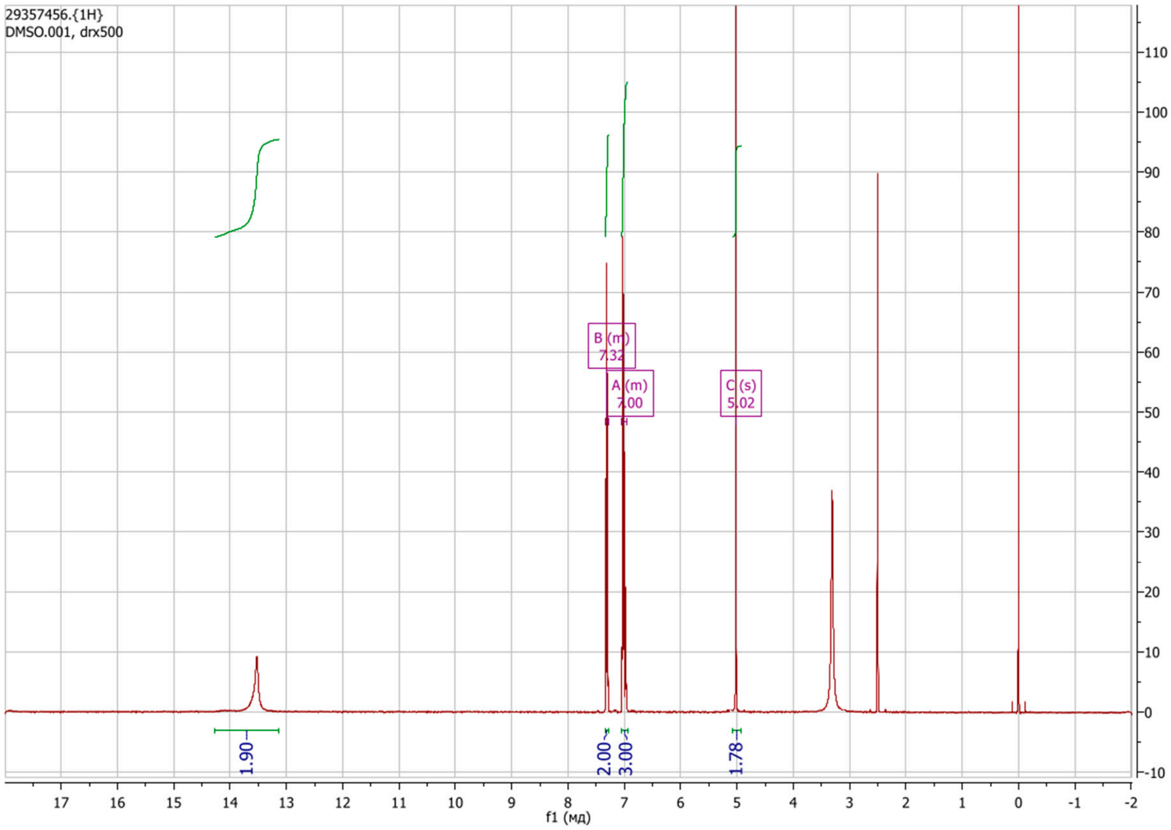

Figure S12. ^{1}H NMR spectrum of 2g (DMSO-d_6)

(2h) 4-ethyl-5-(4-nitrophenyl)-2,4-dihydro-3*H*-1,2,4-triazole-3-thione

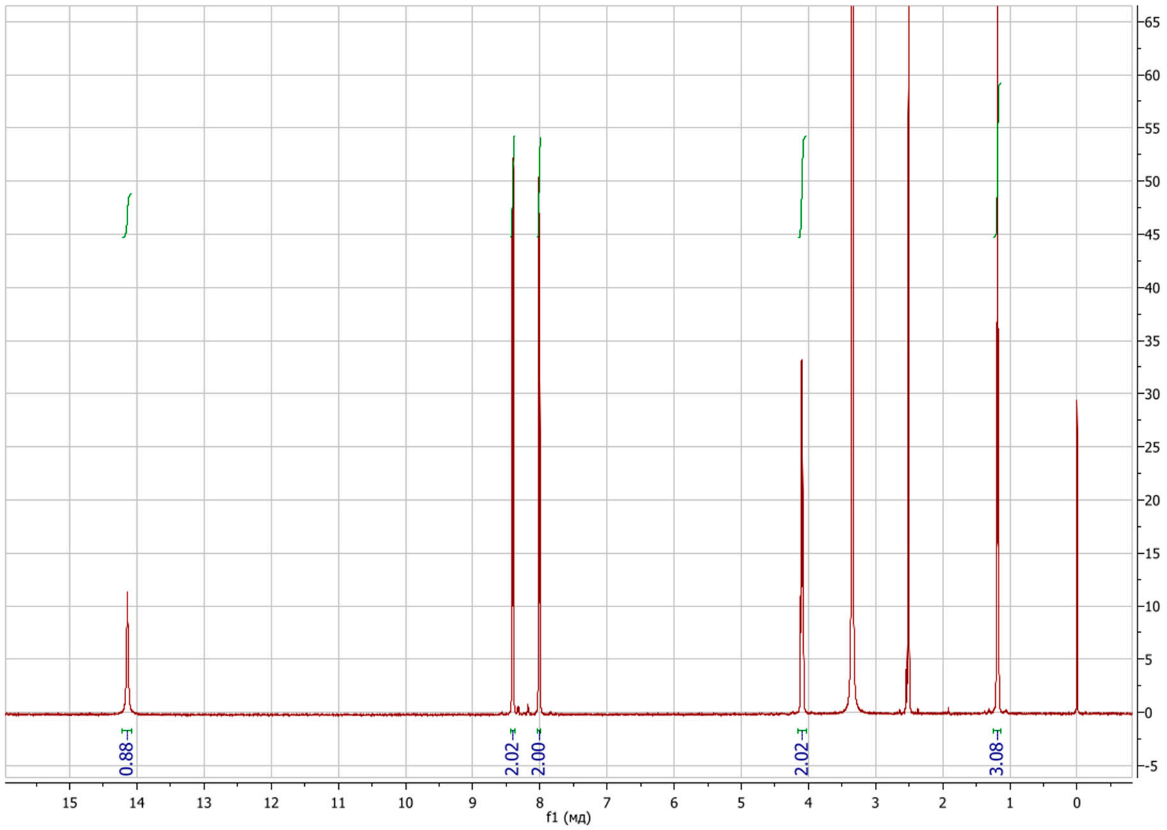

Figure S13. ^{1}H NMR spectrum of 2h (DMSO-d_6)

(2i) 5-(pyridin-3-yl)-2,4-dihydro-3*H*-1,2,4-triazole-3-thione

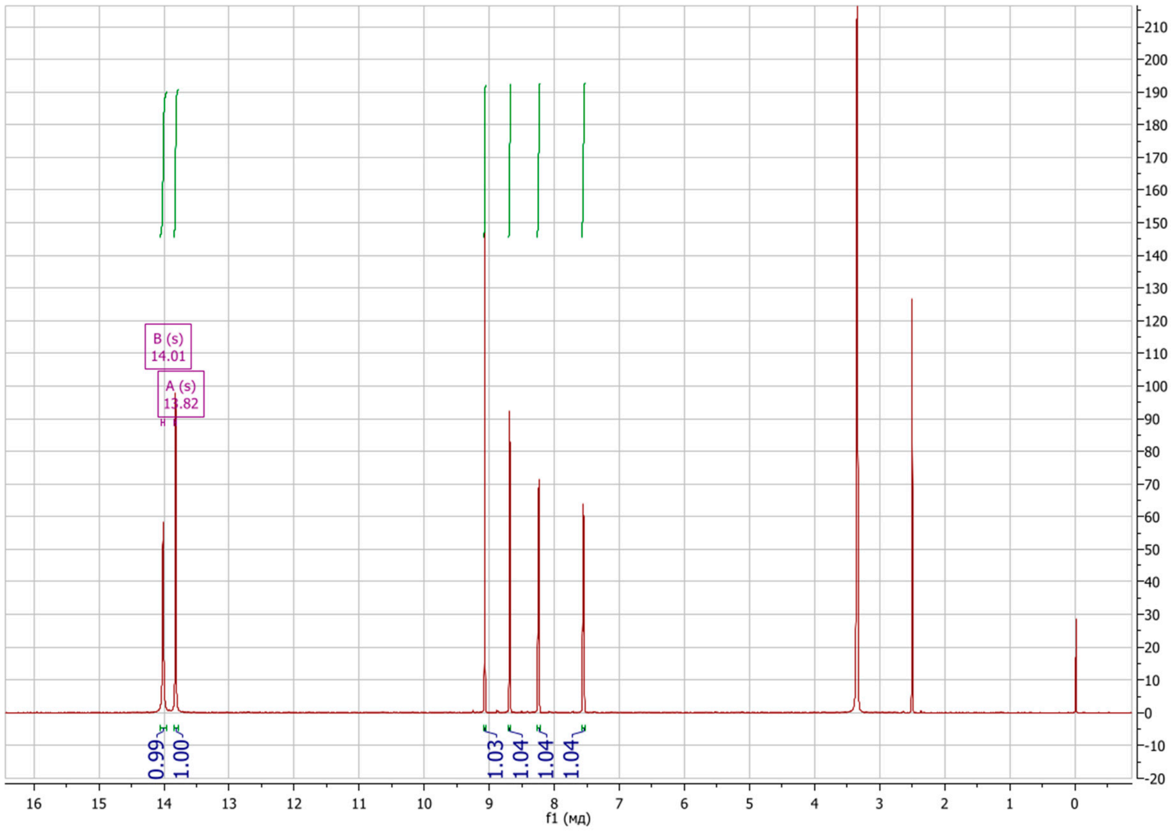

Figure S14. ^{1}H NMR spectrum of 2i (DMSO-d_6)

(2j) 4-ethyl-5-(pyridin-3-yl)-2,4-dihydro-3*H*-1,2,4-triazole-3-thione

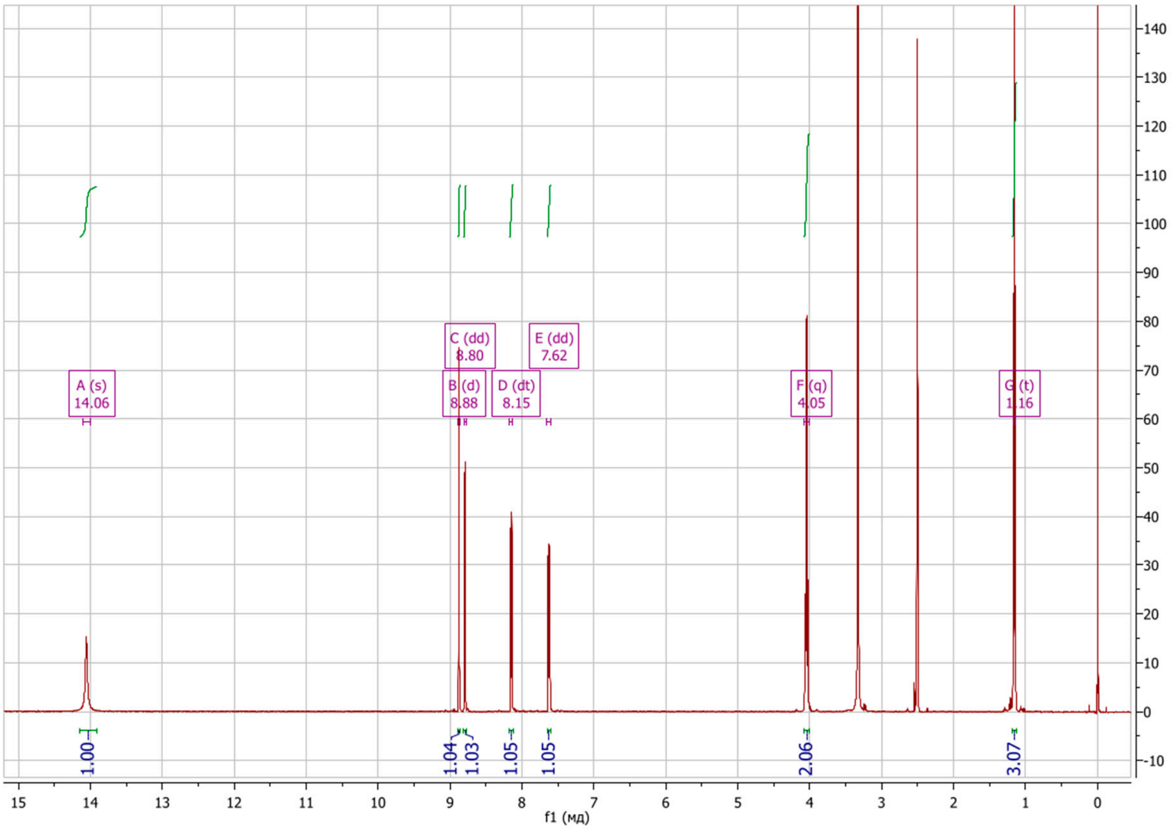

Figure S15. ^{1}H NMR spectrum of 2j (DMSO-d_6)

(2k) 5-[2-(1*H*-benzotriazol-1-yl)ethyl]-2,4-dihydro-3*H*-1,2,4-triazole-3-thione

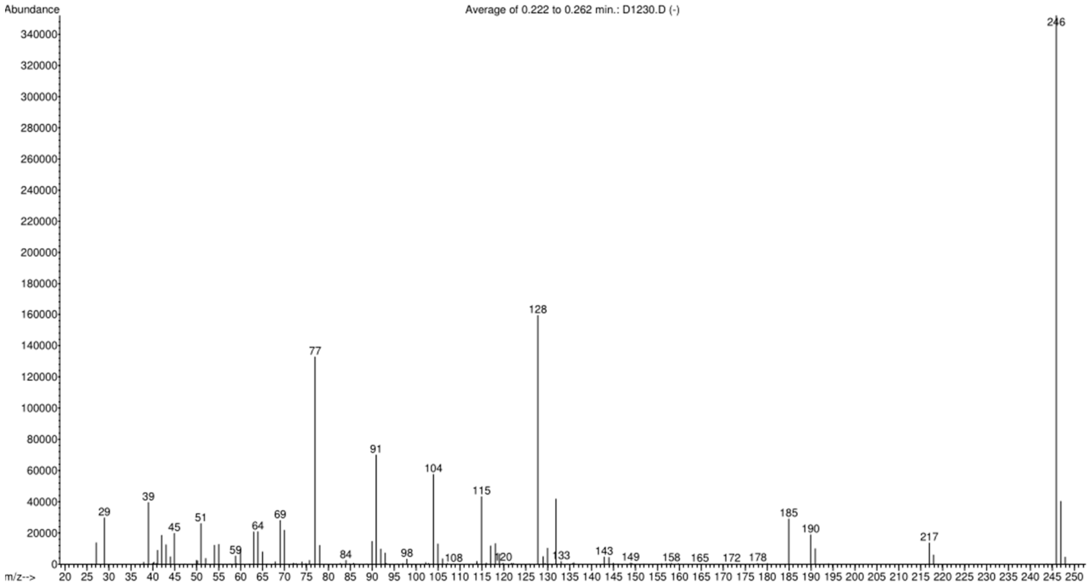

Figure S16. Mass spectrum of 2k

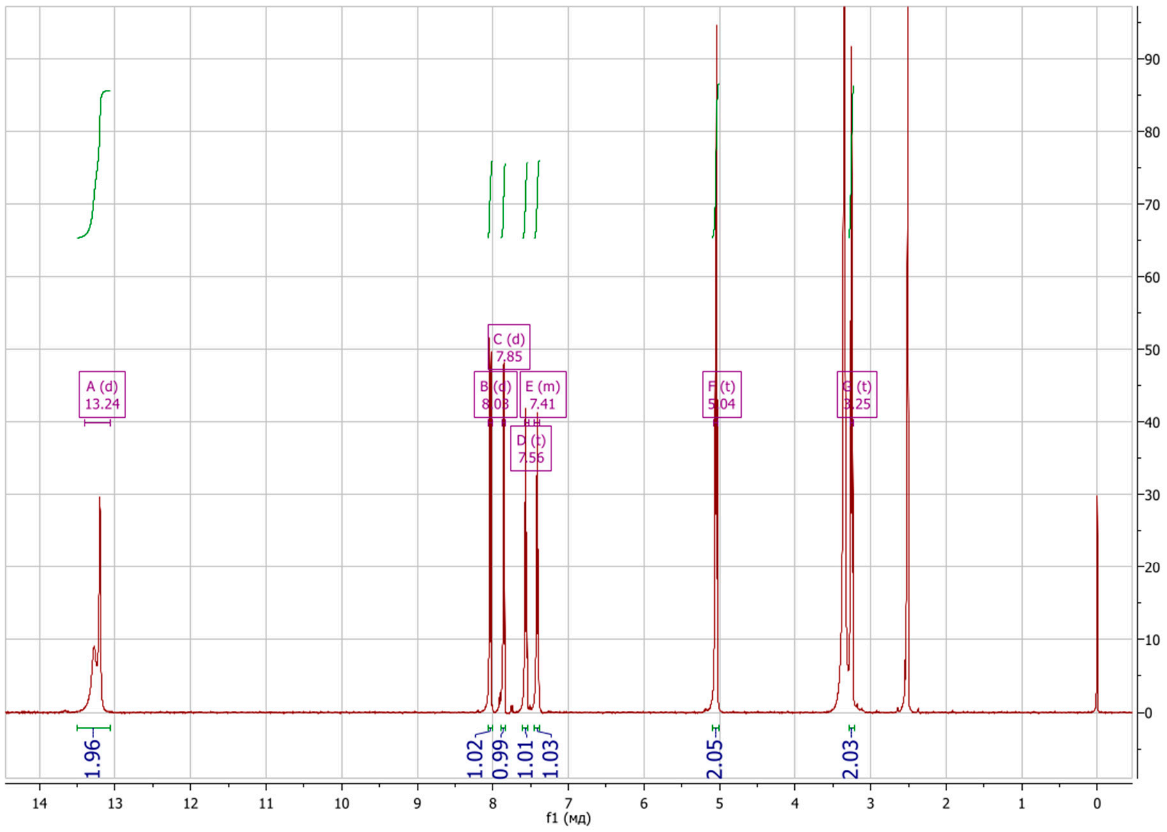

Figure S17. ^{1}H NMR spectrum of 2k (DMSO-d_6)

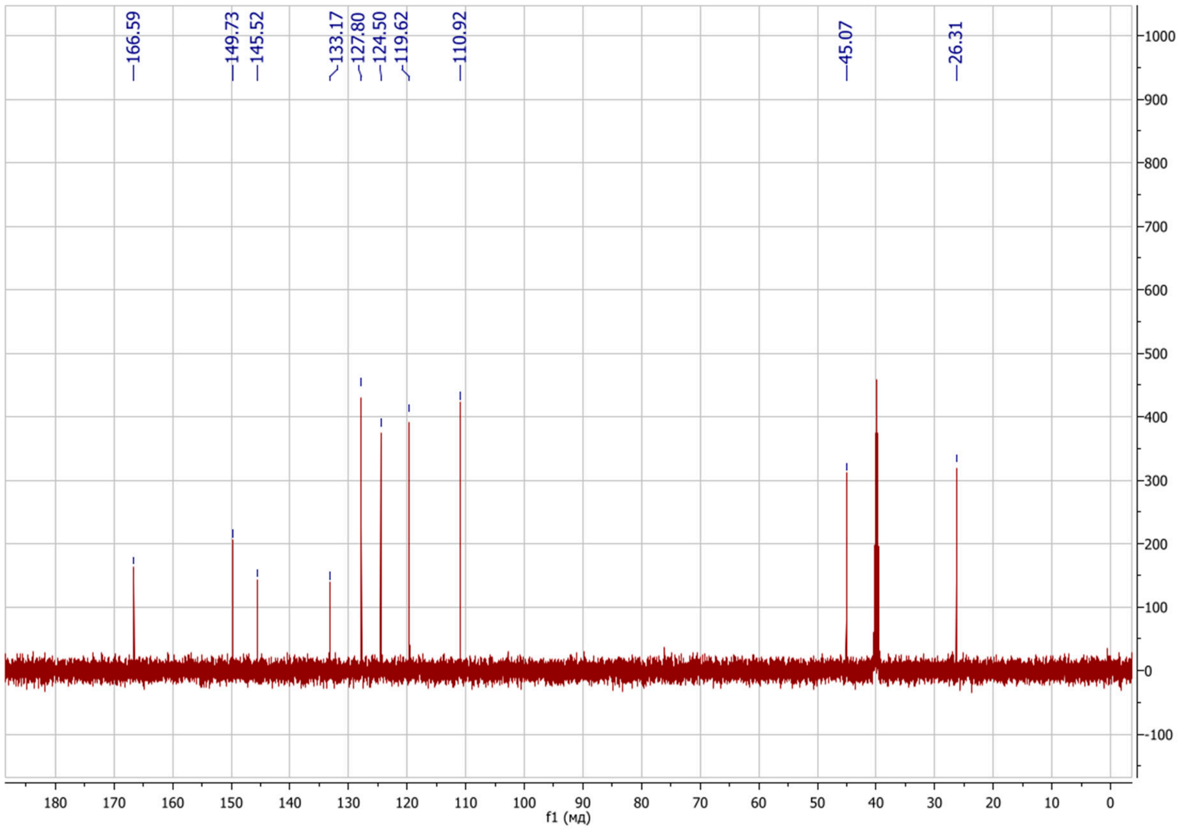

Figure S18. ^{13}C NMR spectrum of 2k (DMSO-d_6)

(2l) 5-[2-(1*H*-benzotriazol-1-yl)ethyl]-4-ethyl-2,4-dihydro-3*H*-1,2,4-triazole-3-thione

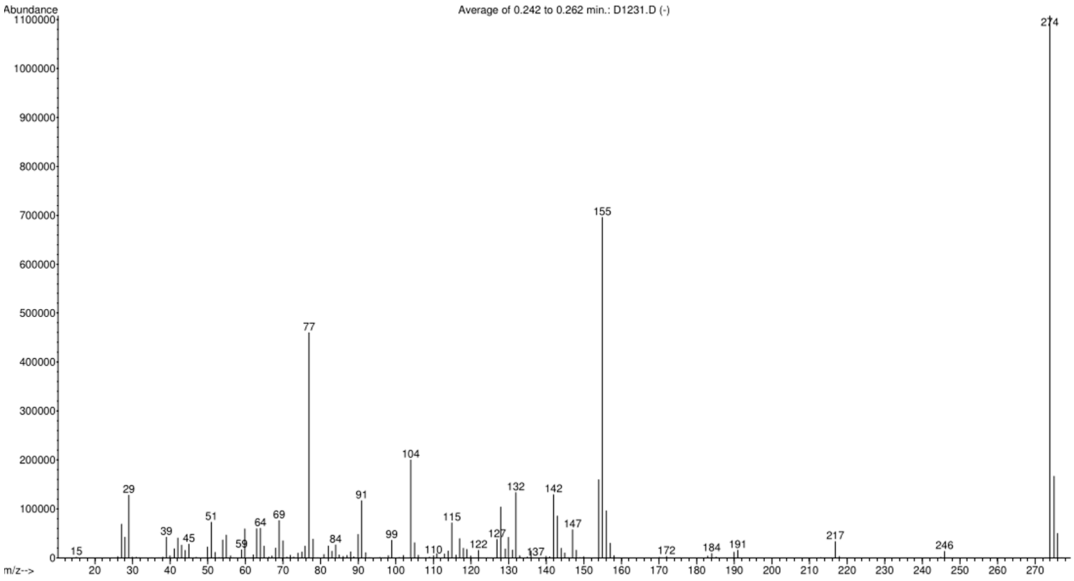

Figure S19. Mass spectrum of 2l

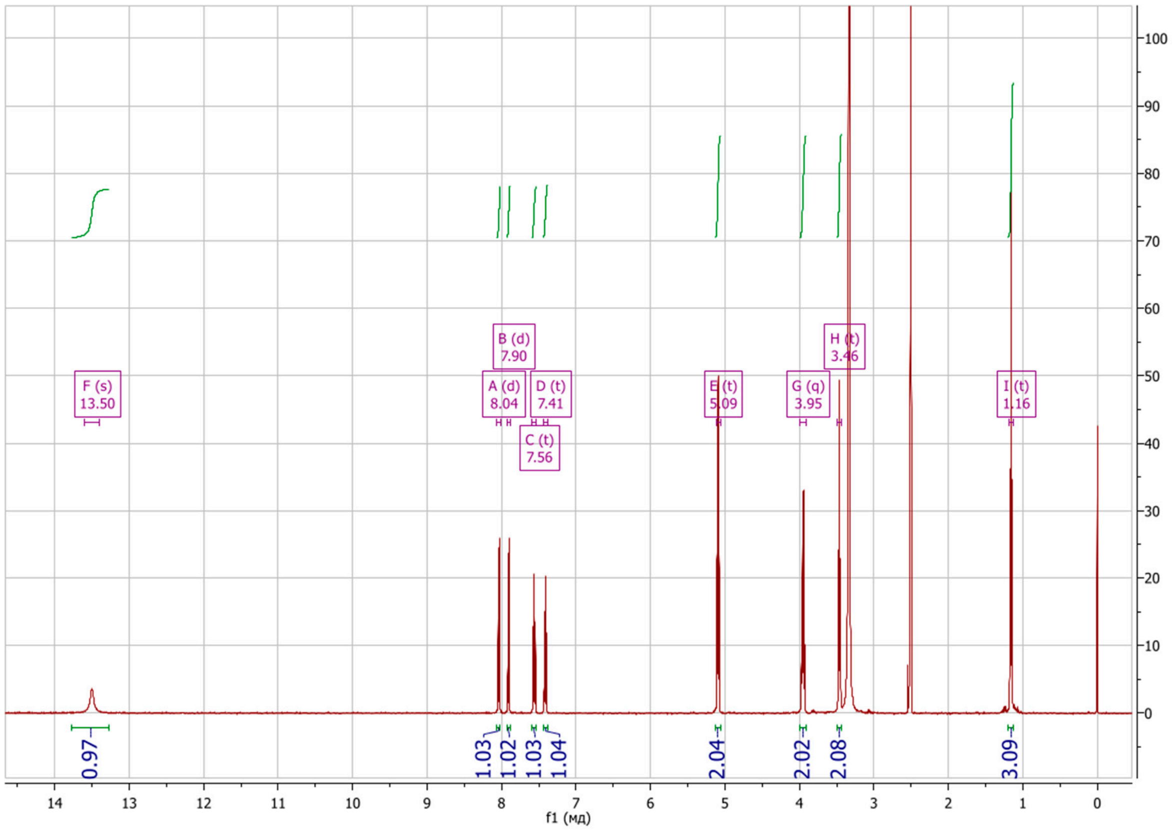

Figure S20. ^{1}H NMR spectrum of 2l (DMSO-d_6)

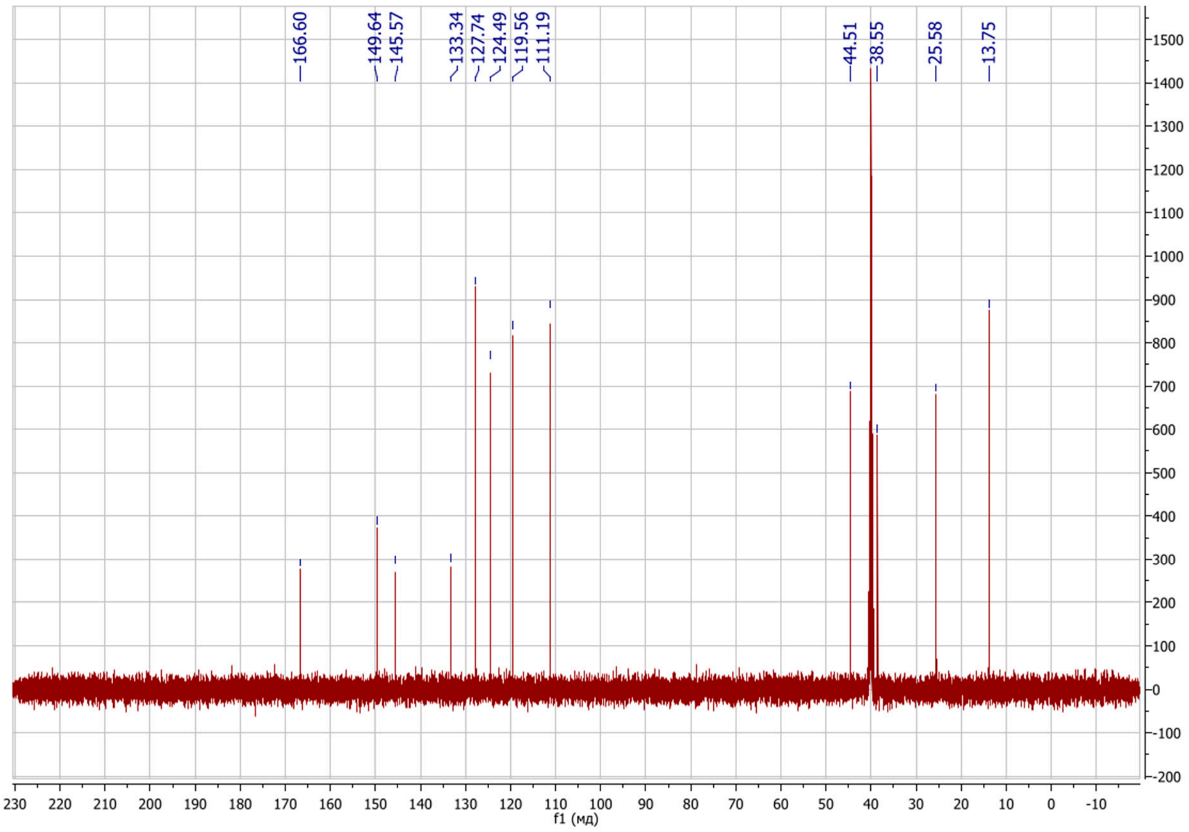

Figure S21. ^{13}C NMR spectrum of 2l (DMSO-d_6)

(2m) 5-[2-(1*H*-benzotriazol-1-yl)ethyl]-4-phenyl-2,4-dihydro-3*H*-1,2,4-triazole-3-thione

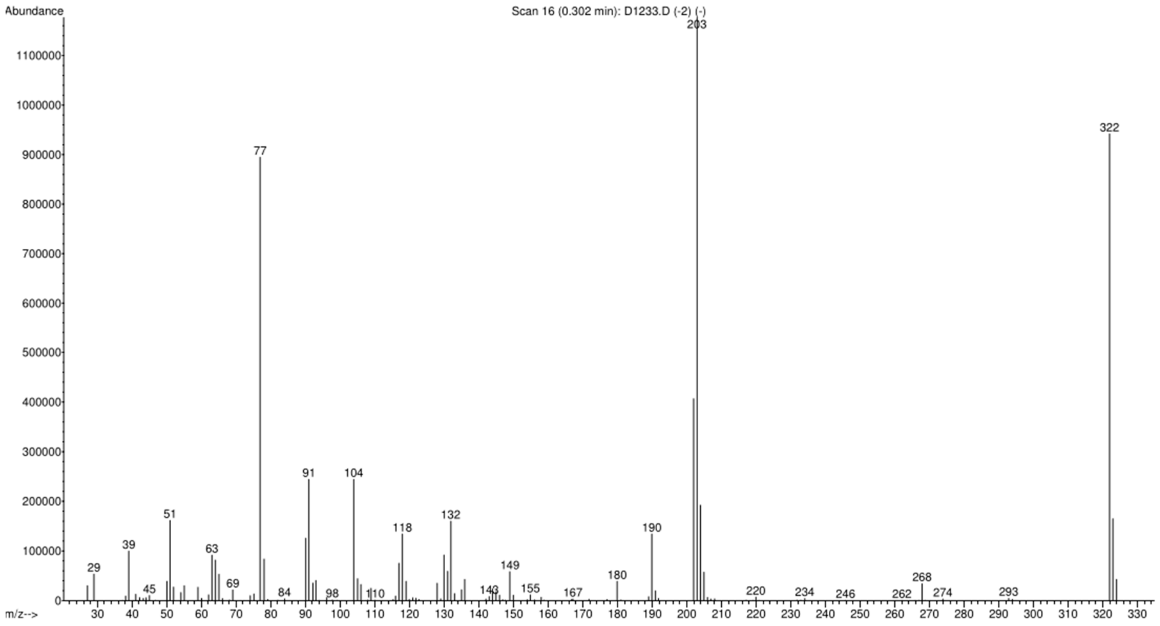

Figure S22. Mass spectrum of 2m

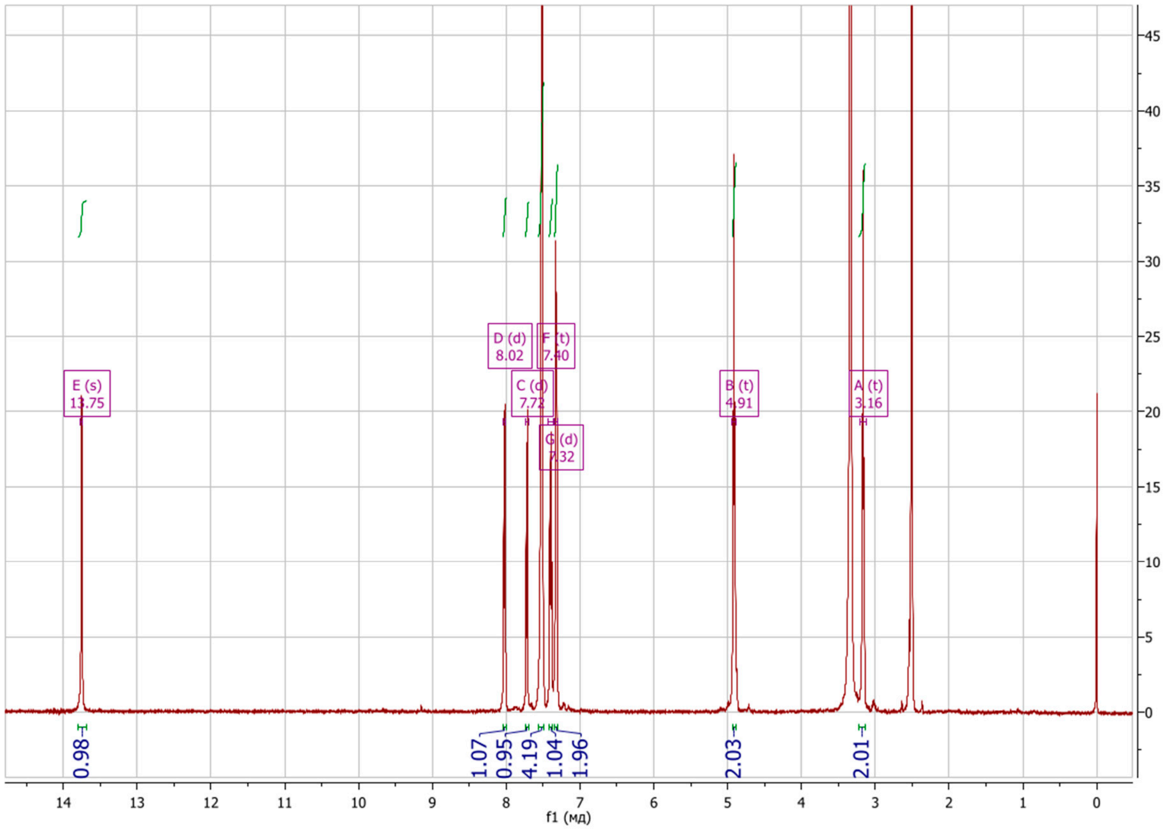

Figure S23. ¹H NMR spectrum of 2m (DMSO-*d*$_6$)

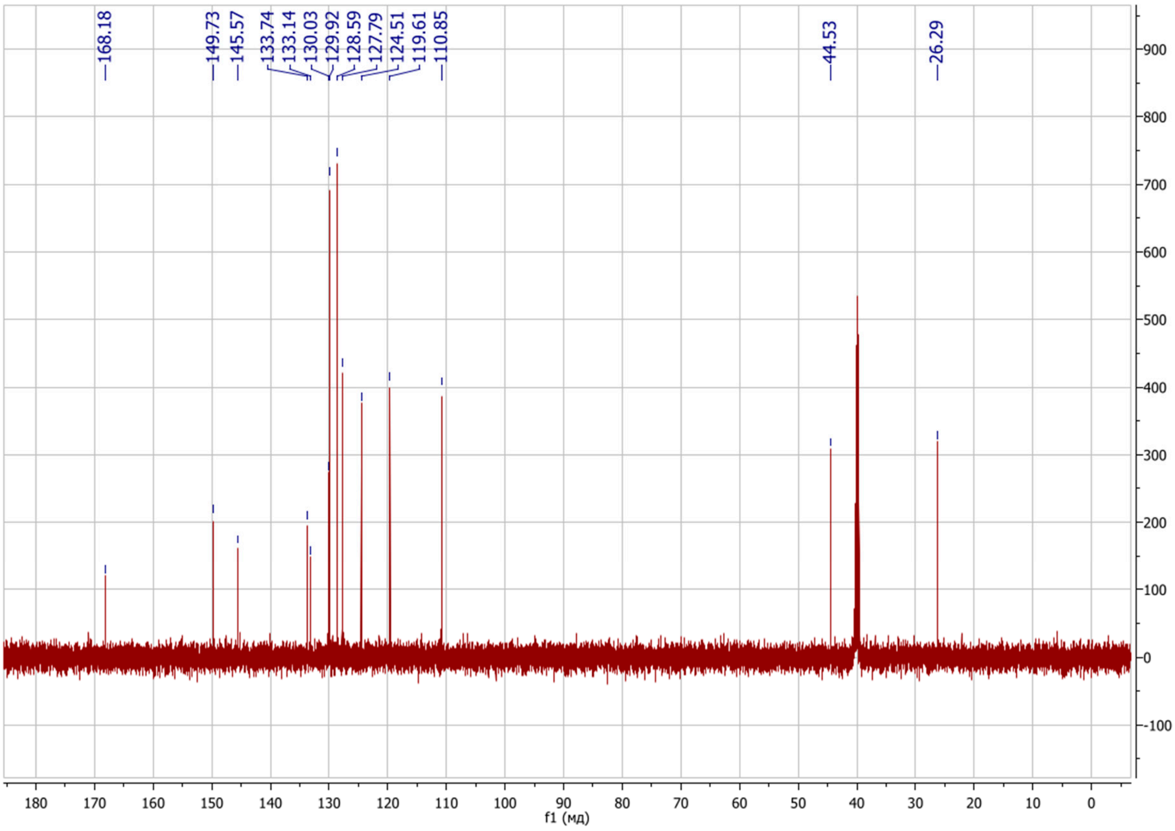

Figure S24. ^{13}C NMR spectrum of 2m (DMSO-d_6)

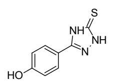

(2o) 5-(4-hydroxyphenyl)-2,4-dihydro-3H-1,2,4-triazole-3-thione

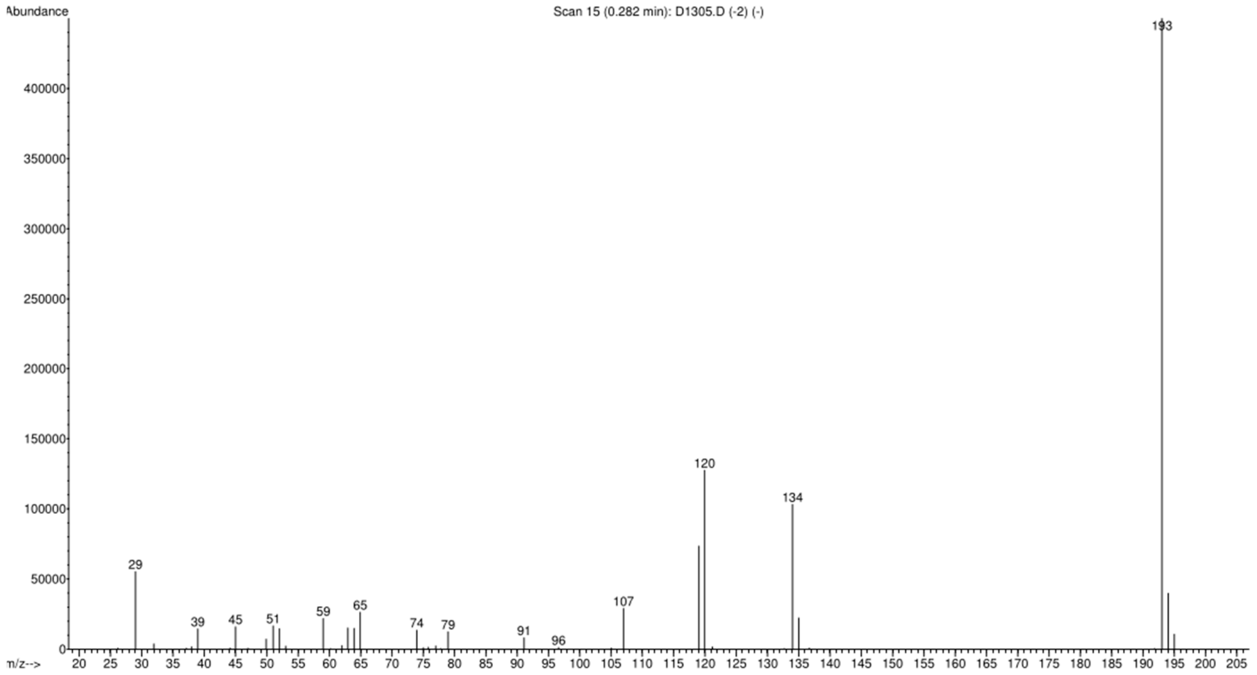

Figure S25. Mass spectrum of 2o

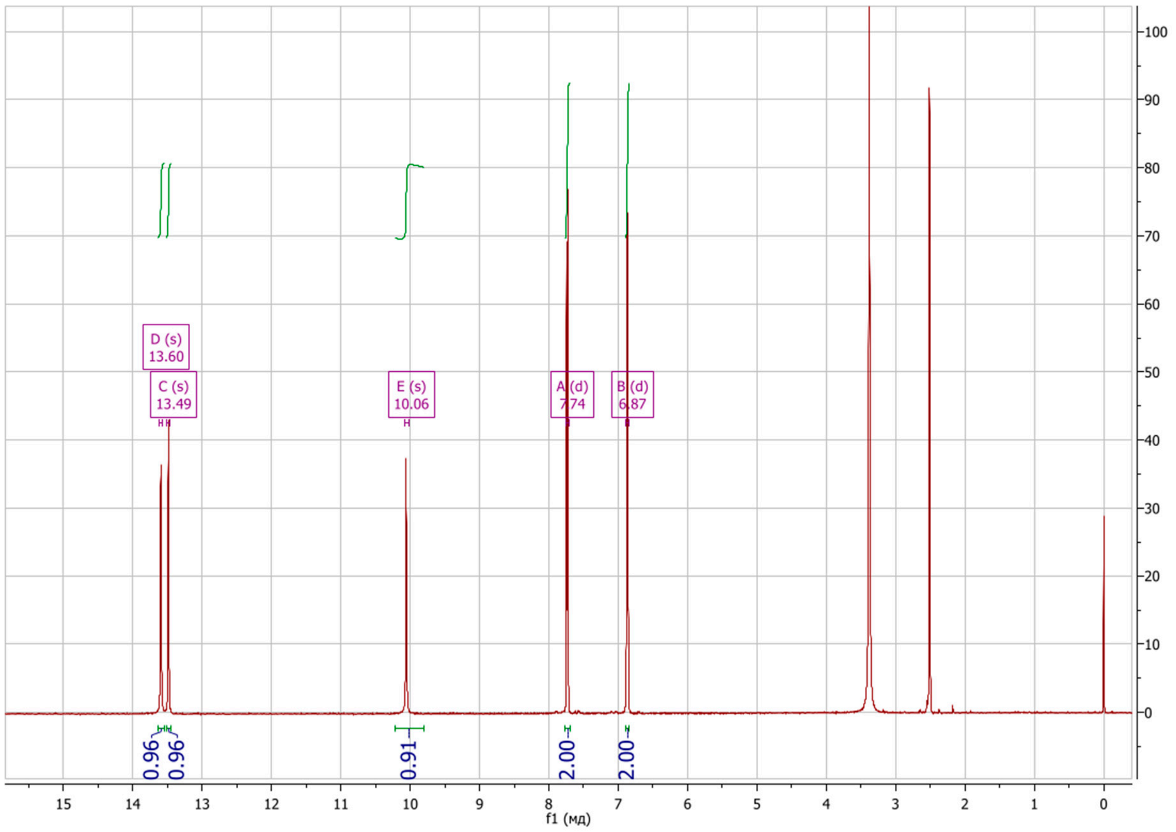

Figure S26. ^{1}H NMR spectrum of 2o (DMSO-d_6)

(2p) 5-[2-(1H-1,2,4-triazol-1-yl)ethyl]-2,4-dihydro-3H-1,2,4-triazole-3-thione

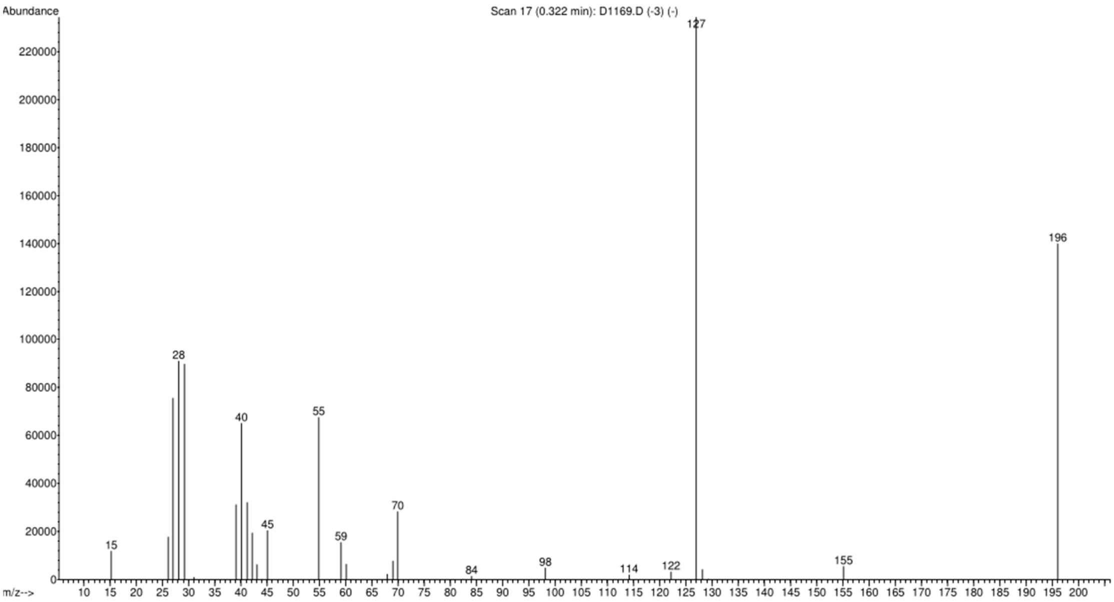

Figure S27. Mass spectrum of 2p

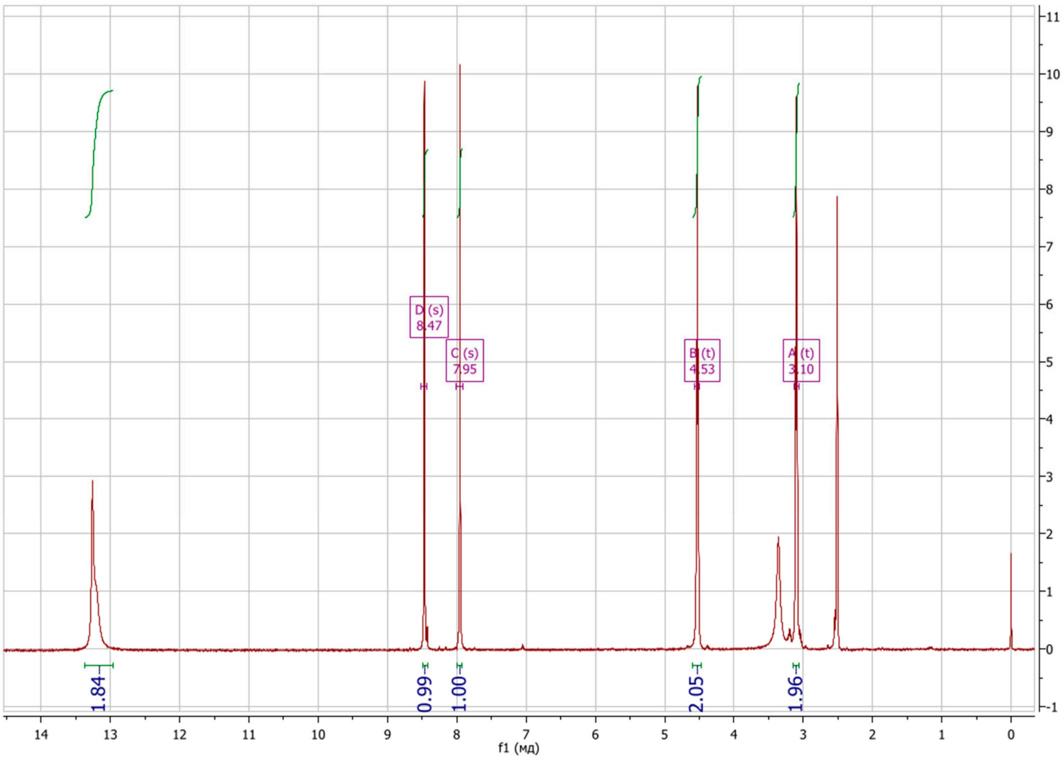

Figure S28. ^{1}H NMR spectrum of 2p (DMSO-d_6)

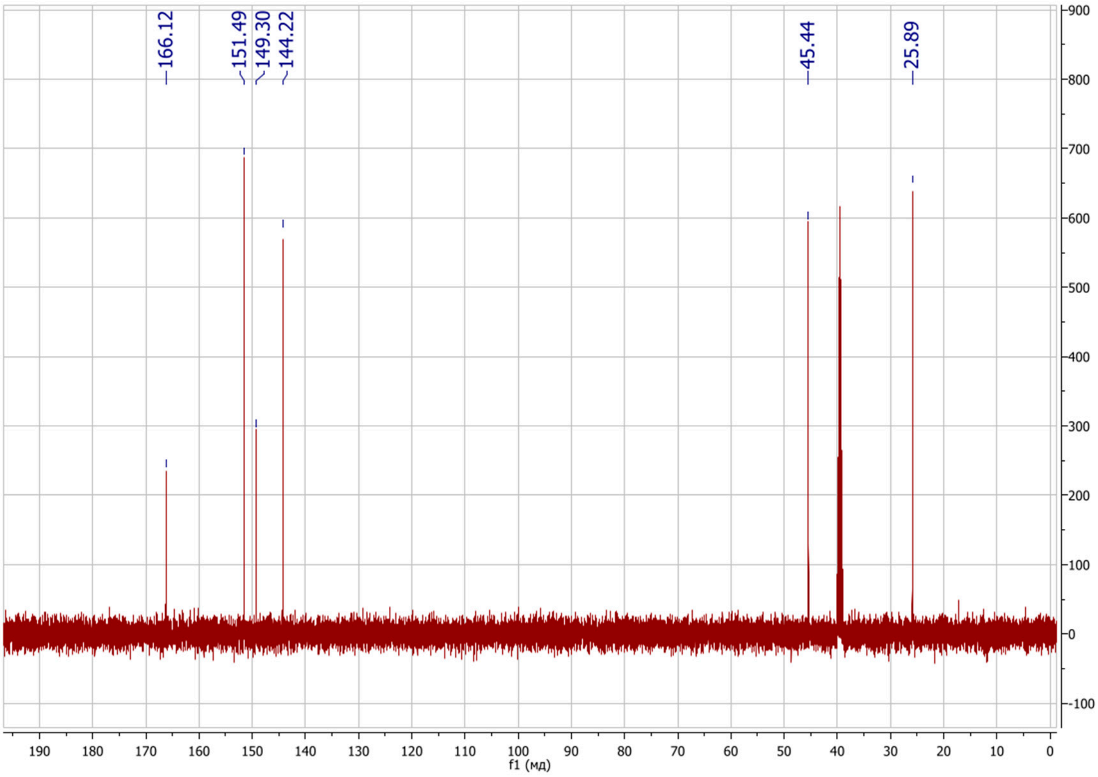

Figure S29. ^{13}C NMR spectrum of 2p (DMSO-d_6)

(3n) *N*-phenyl-5-(2-phenylethyl)-1,3,4-thiadiazol-2-amine

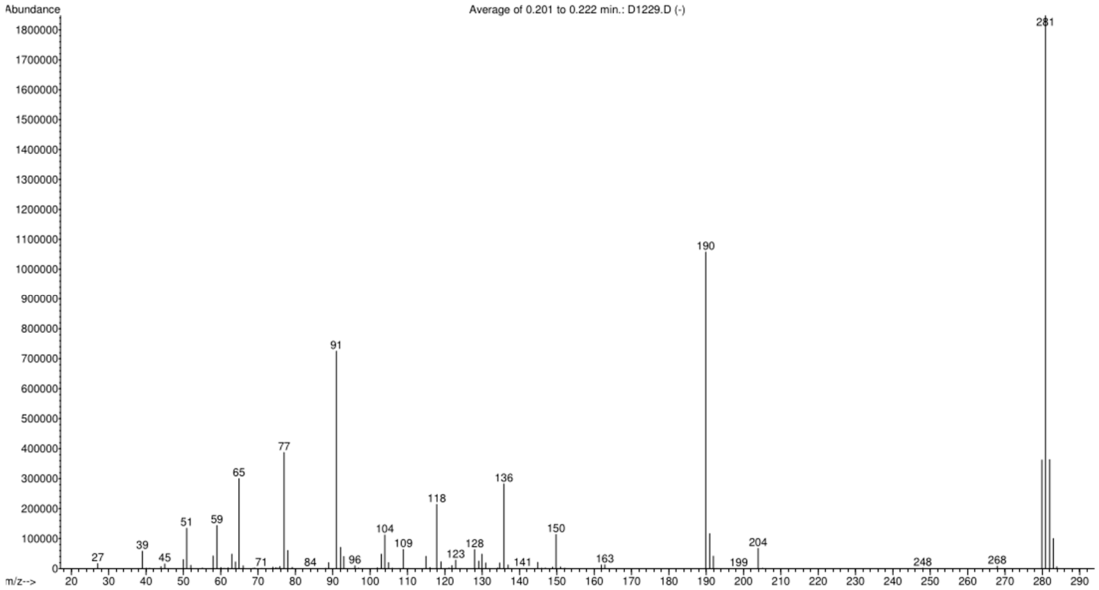

Figure S30. Mass spectrum of 3n

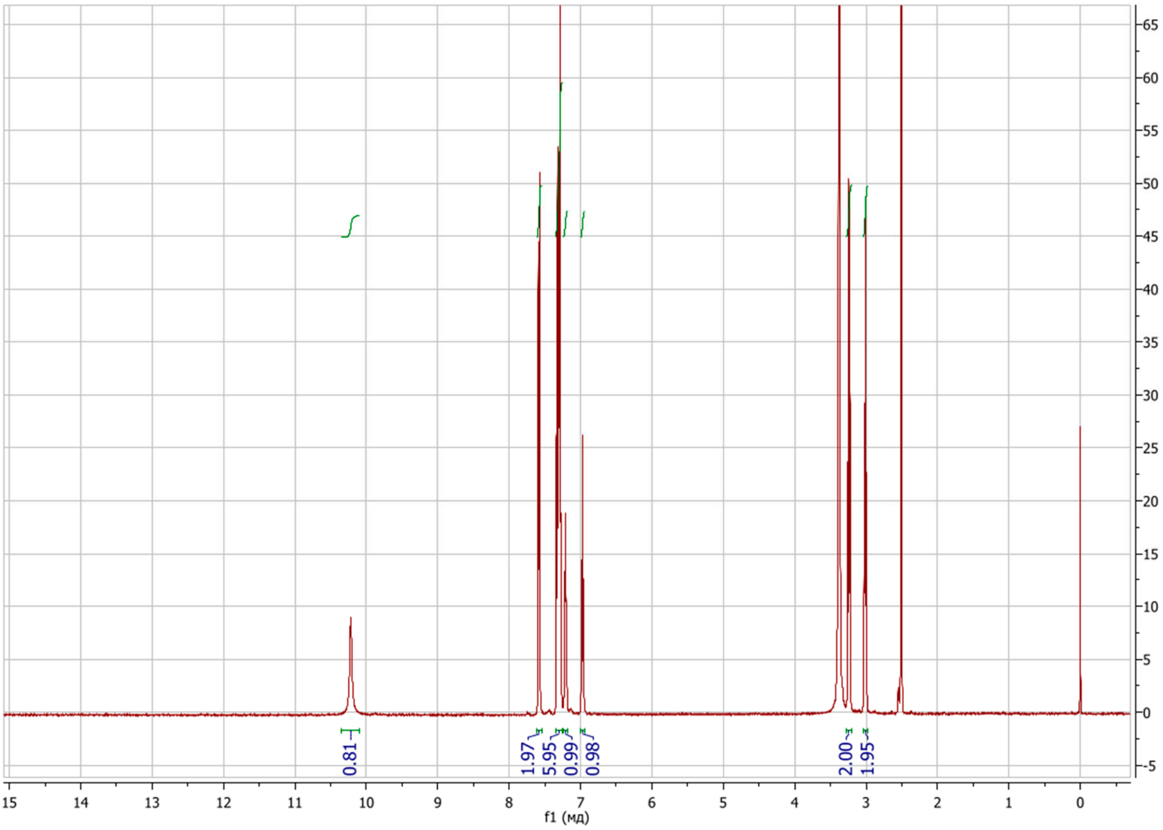

Figure S31. ^{1}H NMR spectrum of 3n (DMSO-d_6)

(3o) 4-(5-amino-1,3,4-thiadiazol-2-yl)phenol

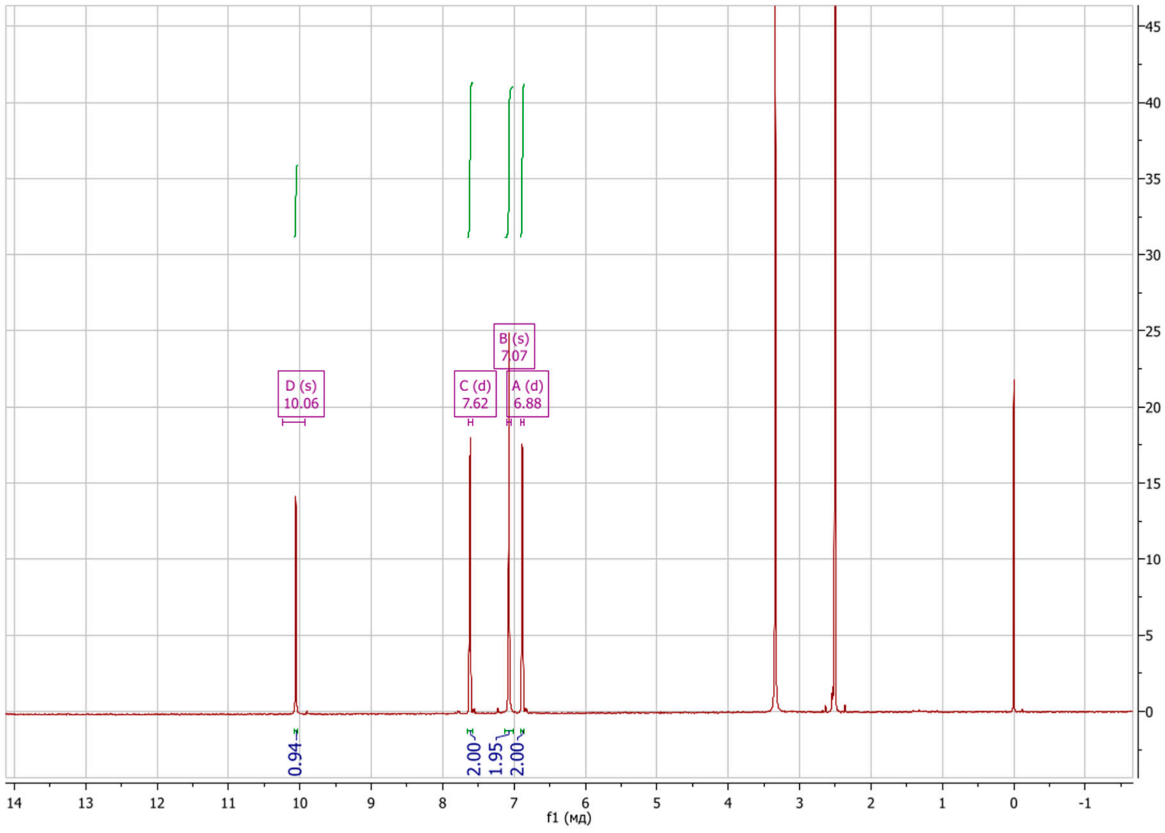

Figure S32. ^{1}H NMR spectrum of 3o (DMSO-d_6)

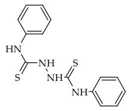

(4) N^1,N^2-diphenylhydrazine-1,2-dicarbothioamide

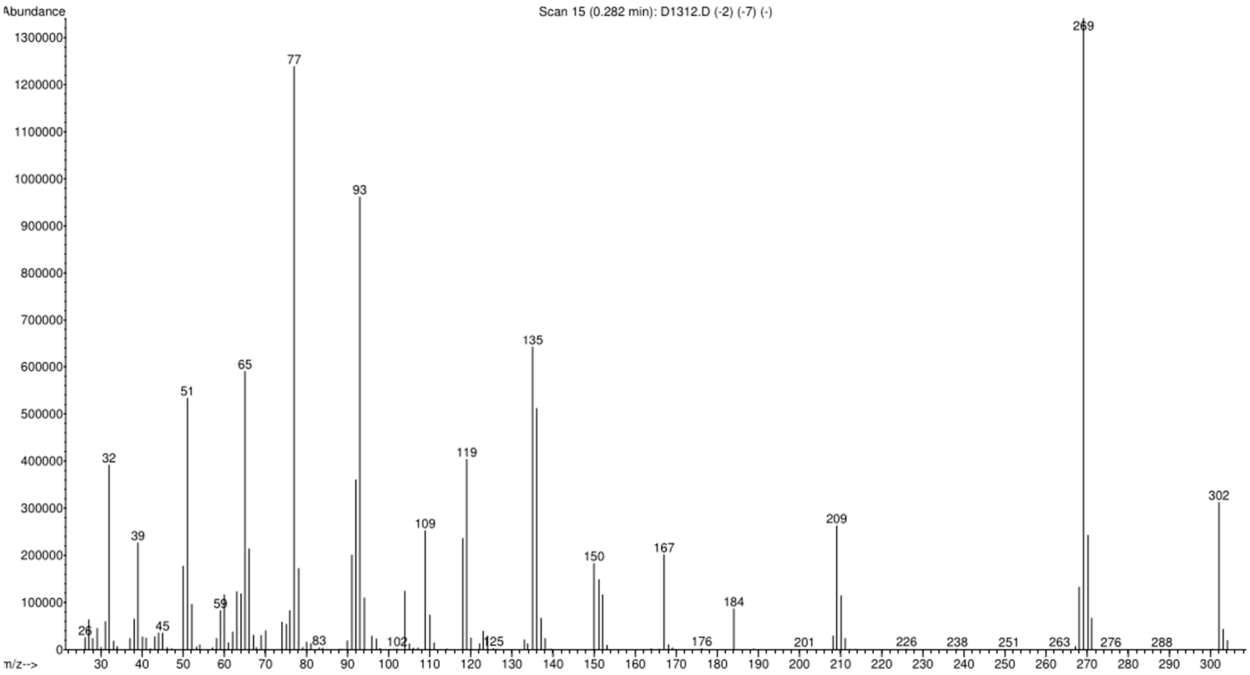

Figure S33. Mass spectrum of 4

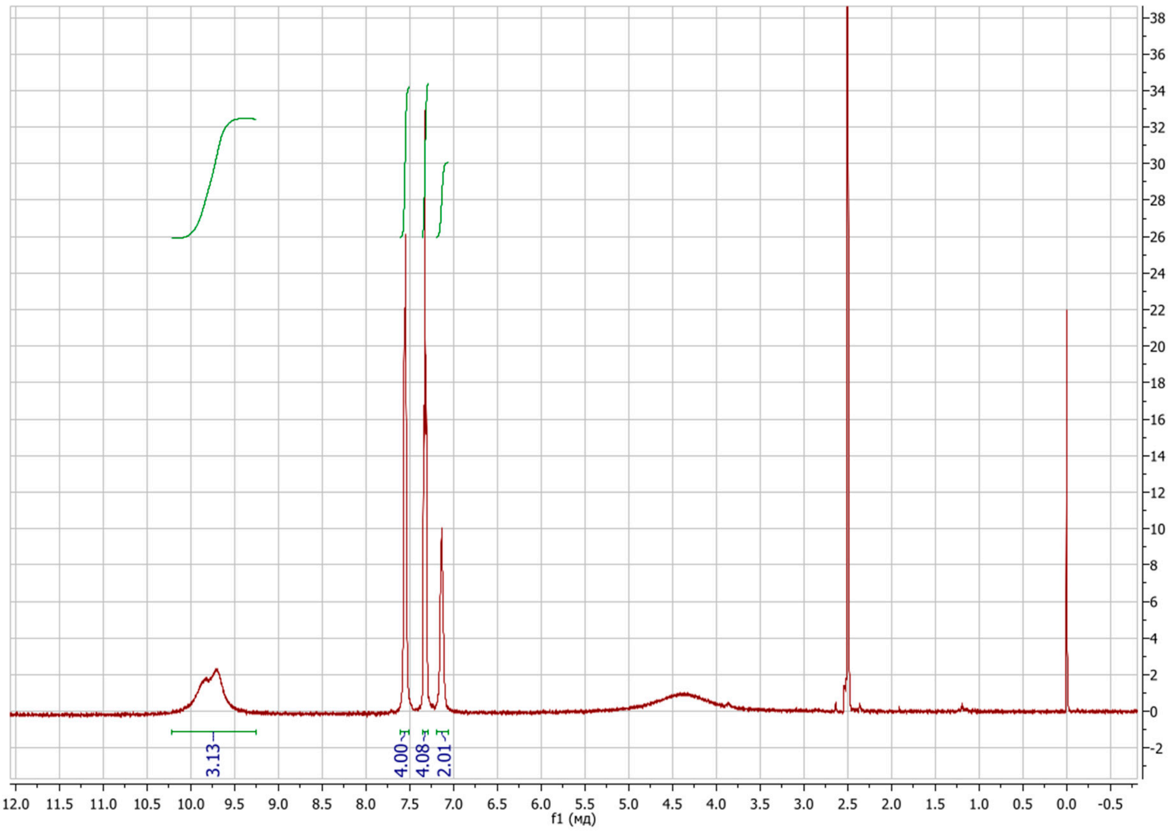

Figure S34. ^{1}H NMR spectrum of 4 (DMSO-d_6)

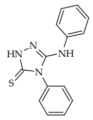

(5) 5-anilino-4-phenyl-2,4-dihydro-3*H*-1,2,4-triazole-3-thione

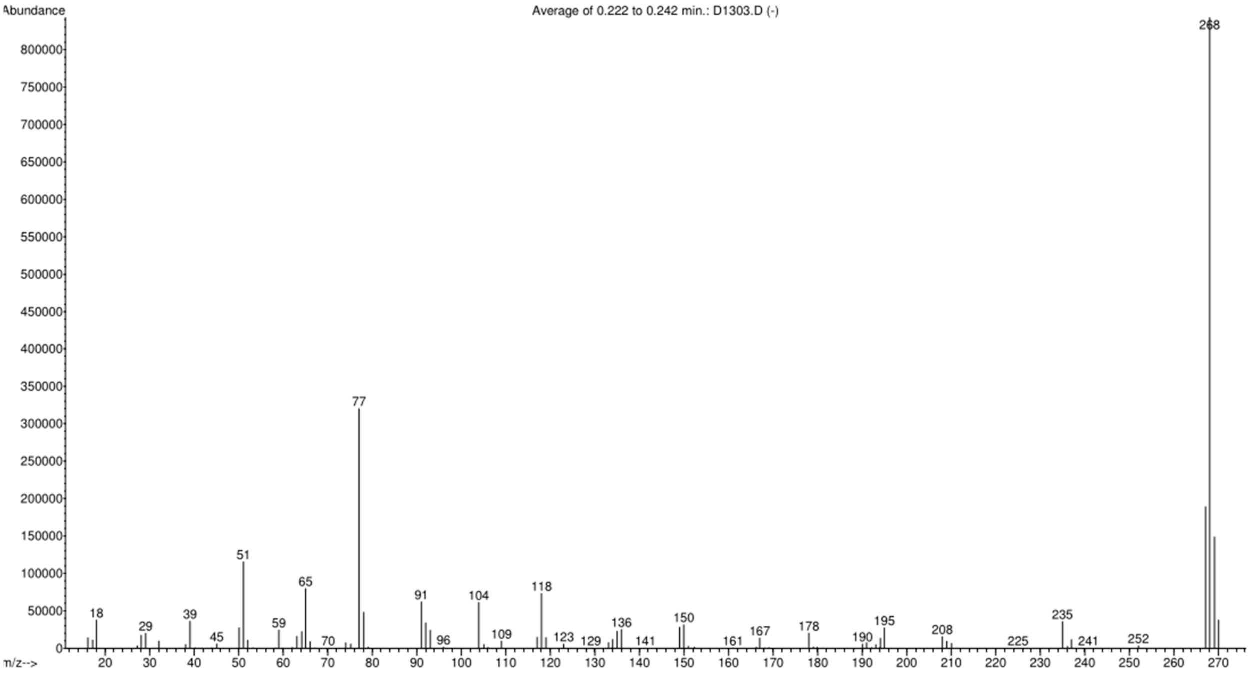

Figure S35. Mass spectrum of 5

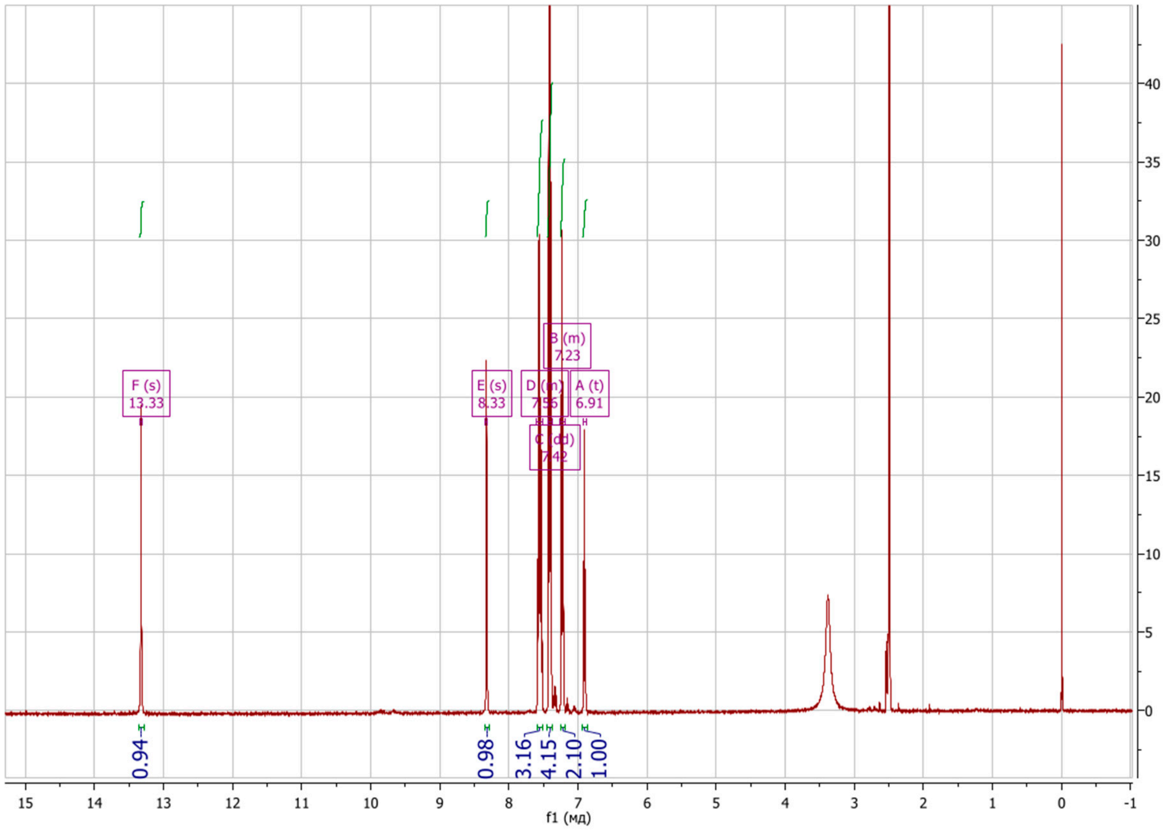

Figure S36. ^1H NMR spectrum of 5 (DMSO-d_6)

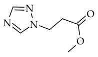

(6) methyl 3-(1*H*-1,2,4-triazol-1-yl)propanoate

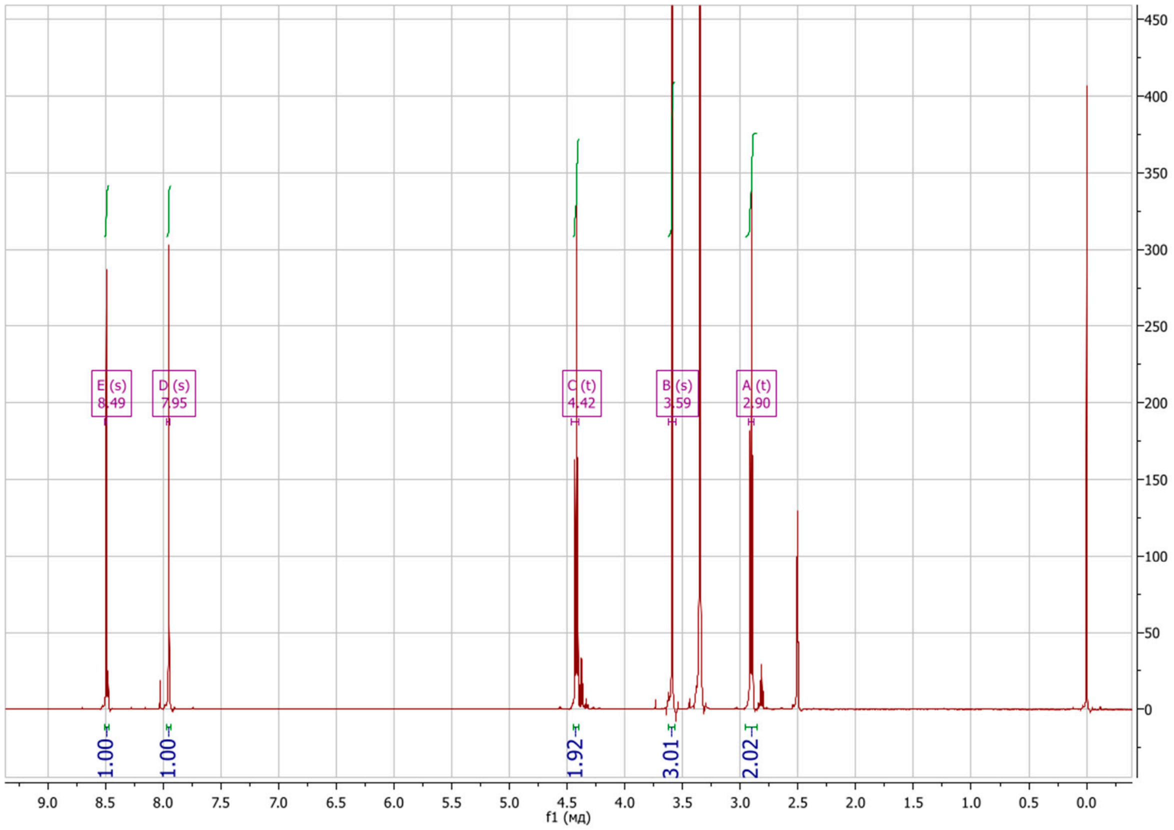

Figure S37. ^1H NMR spectrum of 6 (DMSO-d_6)

Synthesis of 6

3-(1H-1,2,4-Triazol-1-yl)propanoic acid (10 g) was added to a mixture of methanol (50 mL) and concentrated H_2SO_4 (7 mL). The resulting reaction mixture was stirred at room temperature for 24 hours. Methanol was then removed under reduced pressure, water (50 mL) was added to the residue, followed by neutralization with $NaHCO_3$ to pH 6. The mixture was extracted with chloroform (50 mL). The chloroform layer was separated, washed with water (2 × 30 mL), dried over anhydrous Na_2SO_4, and concentrated under vacuum. Yield: 6.1 g (55%).

Attempted synthesis of 3-(1H-1,2,4-triazol-1-yl)propanehydrazide
Method A (ethanol solvent)

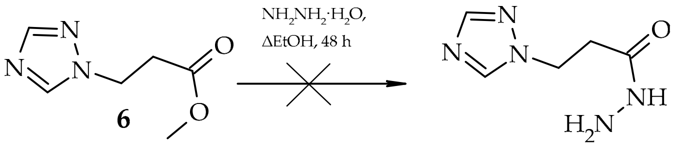

Methyl 3-(1H-1,2,4-triazol-1-yl)propanoate (2.5 g, 16.1 mmol) was added to a mixture of hydrazine monohydrate (1.6 g) in ethanol (10 mL). The reaction mixture was heated under reflux for 48 hours. After cooling to room temperature, the solution was stored in a freezer overnight. No crystallization of the product was observed.

Attempted synthesis of 3-(1H-1,2,4-triazol-1-yl)propanehydrazide
Method B (isopropanol solvent)

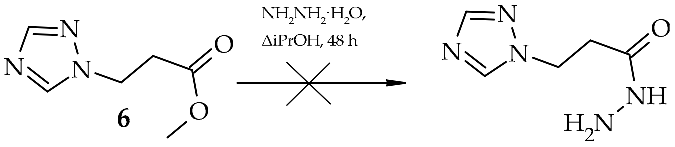

Methyl 3-(1H-1,2,4-triazol-1-yl)propanoate (2.5 g, 16.1 mmol) was added to a mixture of hydrazine monohydrate (1.6 g) in isopropanol (10 mL). The reaction mixture was heated under reflux for 48 hours. After cooling to room temperature, the solution was stored in a freezer overnight. No crystallization of the product was observed.
